# Electrolyte Reactivity at the Charged Ni-Rich Cathode Interface and Degradation in Li-Ion Batteries

**DOI:** 10.1021/acsami.1c22812

**Published:** 2022-03-08

**Authors:** Wesley M. Dose, Israel Temprano, Jennifer P. Allen, Erik Björklund, Christopher A. O’Keefe, Weiqun Li, B. Layla Mehdi, Robert S. Weatherup, Michael F. L. De Volder, Clare P. Grey

**Affiliations:** † Department of Engineering, University of Cambridge, 17 Charles Babbage Road, CB3 0FS Cambridge, U.K.; ‡ Department of Chemistry, University of Cambridge, Lensfield Road, Cambridge CB2 1EW, U.K.; § The Faraday Institution, Quad One, Harwell Science and Innovation Campus, Didcot OX11 0RA, U.K.; ∥ Department of Materials, University of Oxford, Parks Road, Oxford OX1 3PH, U.K.; ⊥ Department of Mechanical, Materials and Aerospace Engineering, University of Liverpool, Liverpool L69 3GH, U.K.

**Keywords:** lithium-ion batteries, degradation, Ni-rich cathode, NMC, electrolyte reactivity, ethylene carbonate, ethyl methyl carbonate, lattice oxygen

## Abstract

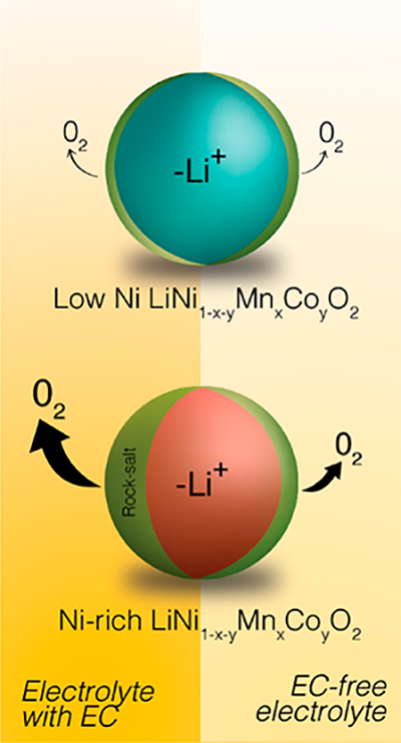

The chemical and electrochemical reactions at the positive electrode–electrolyte interface in Li-ion batteries are hugely influential on cycle life and safety. Ni-rich layered transition metal oxides exhibit higher interfacial reactivity than their lower Ni-content analogues, reacting via mechanisms that are poorly understood. Here, we study the pivotal role of the electrolyte solvent, specifically cyclic ethylene carbonate (EC) and linear ethyl methyl carbonate (EMC), in determining the interfacial reactivity at charged LiNi_0.33_Mn_0.33_Co_0.33_O_2_ (NMC111) and LiNi_0.8_Mn_0.1_Co_0.1_O_2_ (NMC811) cathodes by using both single-solvent model electrolytes and the mixed solvents used in commercial cells. While NMC111 exhibits similar parasitic currents with EC-containing and EC-free electrolytes during high voltage holds in NMC/Li_4_Ti_5_O_12_ (LTO) cells, this is not the case for NMC811. Online gas analysis reveals that the solvent-dependent reactivity for Ni-rich cathodes is related to the extent of lattice oxygen release and accompanying electrolyte decomposition, which is higher for EC-containing than EC-free electrolytes. Combined findings from electrochemical impedance spectroscopy (EIS), TEM, solution NMR, ICP, and XPS reveal that the electrolyte solvent has a profound impact on the degradation of the Ni-rich cathode and the electrolyte. Higher lattice oxygen release with EC-containing electrolytes is coupled with higher cathode interfacial impedance, a thicker oxygen-deficient rock-salt surface reconstruction layer, more electrolyte solvent and salt breakdown, and higher amounts of transition metal dissolution. These processes are suppressed in the EC-free electrolyte, highlighting the incompatibility between Ni-rich cathodes and conventional electrolyte solvents. Finally, new mechanistic insights into the chemical oxidation pathways of electrolyte solvents and, critically, the knock-on chemical and electrochemical reactions that further degrade the electrolyte and electrodes curtailing battery lifetime are provided.

## Introduction

Layered lithium transition metal oxides with the general formula LiNi_1–*x*–*y*_Mn_*x*_Co_*y*_O_2_ (referred to as NMC) are widely used as the positive electrode material in commercial lithium-ion batteries (LIBs) and are the focus of intense ongoing research. Two main strategies are actively pursued to increase the energy density, decrease the cost (in terms of $/kWh), and improve the sustainability of LIBs: charging to higher voltages to extract more capacity from the cathode and/or increasing the Ni content of the cathode material.^1,2^ Problematically, both lead to poorer capacity retention upon cycling.^1,3^ Previous work has shown that delithiation of NMC beyond a certain state of charge (SOC) destabilizes the layered structure resulting in oxygen loss from the near-surface region of the particles and the formation of a spinel and/or rock salt-like reduced surface layer (ReSL).^4−7^ For Ni-rich NMCs, such as LiNi_0.8_Mn_0.1_Co_0.1_O_2_ (NMC811), this occurs at lower potentials vs Li/Li^+^ compared to materials with lower Ni content, such as LiNi_0.33_Mn_0.33_Co_0.33_O_2_ (NMC111).^6,7^ Poorer ionic transport through the ReSL, which evolves structurally and compositionally with cycling, is believed to be a major contributor to impedance rise.^5,8,9^ Oxygen release from NMC is reported to be accompanied by enhanced decomposition of the electrolyte solvent(s),^6,7,10,11^ which are typically cyclic ethylene carbonate (EC) and one or more linear carbonate, e.g., dimethyl carbonate (DMC), ethyl methyl carbonate (EMC), and/or diethyl carbonate (DEC). Release of reactive oxygen species (ROS; e.g., singlet oxygen, ^1^O_2_) from charged NMC has been reported, these species chemically oxidizing the electrolyte solvent(s).^11,12^ Electrolyte solvent breakdown is detrimental to the long-term performance of the cell in a number of ways, including: depletion of the electrolyte; evolution of gases (i.e., CO and CO_2_) that can cause swelling and/or further reactions at either electrode;^13−15^ deposition of decomposition products on the NMC particles yielding the cathode electrolyte interphase (CEI);^16,17^ formation of soluble decomposition species (including acidic species) that may further react leading to additional degradation of the electrolyte; transition metal (TM) dissolution from the cathode, and disruption of the solid electrolyte interphase (SEI) on the anode.^18−21^ Therefore, understanding the electrochemical and chemical reactions at the electrolyte-electrode interface (EEI) of Ni-rich NMCs is of paramount importance to increase the energy density, cycle life, and safety of LIBs.

The pivotal role of the electrolyte chemistry in determining the long-term battery performance was highlighted in work by Dahn and co-workers.^1,22^ They demonstrated that electrolytes free of EC gave a remarkable improvement to the high voltage (4.4 V) cyclability of LiNi_0.5_Mn_0.3_Co_0.2_O_2_/graphite full cells. Following this, Manthiram and co-workers recently reported a similar improvement for Ni-rich LiNi_0.94_Co_0.06_O_2_/graphite full cells with EC-free electrolytes, showing fewer CEI deposits on the NMC particles, less TM dissolution/deposition, and improved cathode bulk structural reversibility compared to conventional EC-containing electrolytes.^2^ Gasteiger and co-workers have proposed that the observed performance enhancement in EC-free electrolytes arises due to the different stability of cyclic and linear carbonates toward chemical attack of singlet oxygen:^11^ online gas analysis revealed that EC decomposes in the presence of singlet oxygen (produced in this case by photoexcitation of Rose Bengal dye in triplet oxygen saturated solvent) while DMC is stable under these conditions. However, the relative chemical reactivity of cyclic and linear carbonate-based electrolytes at the EEI of NMC cathodes is not yet understood. Furthermore, other important questions remain unanswered; specifically, does the electrolyte solvent change the amount of lattice oxygen loss, and what impact does this have on the degradation of the electrolyte solvent, the electrolyte salt, and the NMC interface?

In this study, we examine the influence of the electrolyte solvent on the parasitic reactions at the EEI for low and high Ni content NMCs during a high-voltage potentiostatic hold. The electrochemical protocol subjects the EEI to prolonged oxidizing conditions, with the current measured by the potentiostat sensitive to the *electrochemical* oxidation of the electrolyte and the loss of lattice oxygen from the NMC.^23^ As mentioned above, the latter is directly related to the *chemical* oxidation of the electrolyte. A NMC/Li_4_Ti_5_O_12_ (LTO) cell pairing is used, since at 1.55 V vs Li/Li^+^, there is expected to be a negligible continuous electrochemical electrolyte reduction at the LTO electrode,^24,25^ thereby avoiding crossover of electrolyte reduction products formed at the anode to the cathode.^26^

To establish the relative interfacial stability of common carbonate solvents, two model, single solvent electrolytes comprised of EC or EMC with LiPF_6_ salt were compared with a conventional electrolyte (LP57) containing both EC and EMC (3:7 by weight) with LiPF_6_ salt. First, the amount of gas evolution from lattice oxygen release and electrolyte breakdown was determined by online electrochemical mass spectrometry (OEMS). After the high-voltage potentiostatic hold protocol, the electrodes and electrolyte were extracted from coin cells for post-test analysis. High-resolution transmission electron microscopy (HRTEM) and X-ray photoelectron spectroscopy (XPS) were used to analyze the surface degradation of the NMC cathode, and these measurements were coupled with three-electrode electrochemical impedance spectroscopy (EIS) to probe the ion transport behavior across the NMC interface. The electrolyte was analyzed by solution nuclear magnetic resonance (NMR) spectroscopy to identify any soluble decomposition products formed. Transition metal dissolution from the NMC cathode was evaluated by inductively coupled plasma-optical emission spectroscopy (ICP-OES). By combining several advanced characterization techniques, we propose reaction pathways and mechanisms for the degradation occurring at the EEI of Ni-rich NMC cathodes with EC-containing and EC-free electrolytes. The important fundamental insights regarding the Ni content-dependent and electrolyte-dependent interfacial reactivity of NMC cathodes provided herein will guide ongoing efforts to stabilize the interface of Ni-rich NMC and to achieve stable long-term battery performance.

## Experimental Section

### Materials and Electrode Fabrication

NMC111 and NMC811 cathodes and LTO anodes were prepared at large-scale in the Cell Analysis, Modeling, and Prototyping (CAMP) facility at Argonne National Laboratory. The cathodes consisted of 90 wt % NMC (NMC111, Toda; NMC811, Targray), 5 wt % polyvinylidene difluoride binder (PVDF, Solvay 5130), and 5 wt % conductive carbon (Timcal C45) cast onto 20 μm thick aluminum foil using *N*-methyl-2-pyrrolidone (NMP) as the solvent. The NMC111 and NMC811 cathode sheets had loadings of 10.10 mg_NMC_ cm^–2^ and 8.21 mg_NMC_ cm^–2^, respectively, corresponding to ∼1.48 mAh cm^–2^ based on 147 mAh g^–1^_NMC_ for NMC111 and ∼1.52 mAh cm^–2^ based on 185 mAh g^–1^_NMC_ for NMC811. The anodes consisted of 87 wt % LTO (Samsung Fine Chemicals), 5 wt % conductive carbon (Timcal C45), and 8 wt % PVDF binder (Kureha 9300) cast onto 20 μm thick aluminum foil using NMP as the solvent. The anode sheets had loadings of 12.27 mg_LTO_ cm^–2^ corresponding to ∼1.96 mAh cm^–2^ based on 160 mAh g^–1^_LTO_. After drying, electrodes were calendered using a heated (80 °C) two-roller hydraulic-driven roll press (A-PRO Co.) to 30% porosity. Circular electrodes with 14 mm (cathode) and 15 mm (anode) diameters were punched and dried at 120 °C for at least 12 h under dynamic vacuum before being transferred to an Ar filled glovebox (<0.5 ppm of O_2_ and H_2_O, MBraun). The electrode capacity balancing of anode and cathode (N/P ratio) was set to ≈1.3:1.0.

NMC111 powder (Toda) was annealed in an alumina crucible at 750 °C for 4 h with a 5 °C min^–1^ heating rate under air atmosphere using a muffle furnace (MTI). After natural cooling, a slurry consisting of 90 wt % annealed NMC111, 5 wt % PVDF binder (PI-KEM), and 5 wt % conductive carbon (C45, PI-KEM) was cast onto 20 μm thick aluminum foil using NMP as the solvent. The annealed NMC111 cathodes had loadings of 8.0 mg_NMC_ cm^–2^ corresponding to ∼1.2 mAh cm^–2^ based on 147 mAh g^–1^_NMC_.

LiMn_2_O_4_ (LMO) cathodes consisted of 90 wt % LMO (Sigma-Aldrich, < 0.5 μm particle size (BET), > 99%), 5 wt % PVDF binder (PI-KEM), and 5 wt % conductive carbon (C45, PI-KEM) cast onto 20 μm thick aluminum foil using NMP as the solvent. The LMO cathodes had loadings of 3.3 mg_LMO_ cm^–2^ corresponding to ∼0.5 mAh cm^–2^ based on 148 mAh g^–1^_LMO_.

The baseline electrolyte was 1 M LiPF_6_ in ethylene carbonate (EC):ethyl methyl carbonate (EMC) 3:7 (by weight, LP57, SoulBrain). Single solvent electrolytes were 1.5 M LiPF_6_ (99.9%, Solvionic) in EC (anhydrous 99%, Sigma-Aldrich) and 1.5 M LiPF_6_ in EMC (99.9%, Solvionic).

### Electrochemical Cell Assembly and Protocols

The 2032-type coin cells (Hohsen) were assembled in a full cell setup with a 14 mm diameter cathode, 15 mm diameter anode, and 16 mm diameter Celgard 2325 separator (PI-KEM) or glass fiber separator (grade GF/A, Whatman) soaked in 40 or 80 μL of electrolyte, respectively. Separators were dried for at least 24 h under dynamic vacuum at 60 or 120 °C, respectively. Three-electrode PAT cells (EL-Cell) were assembled with 18 mm diameter cathode and anode, glass fiber separator (260 μm thickness, grade GF/A) soaked in 100 μL of electrolyte, and a lithium metal ring electrode set in an insulation sleeve (EL-Cell).

After assembly, the cells were charged galvanostatically at C/20 (assuming a practical capacity of 147 mAh g^–1^_NMC_ for NMC111 and 185 mAh g^–1^_NMC_ for NMC811) to 3.05 V (vs LTO) and held at that voltage for 60 h while the current was recorded, after which they were discharged at C/20 to 1.45 V (Biologic VMP3 or BCS 805 series). For simplicity, this protocol is referred to as the “60 h voltage hold (VH) protocol.” Subsequently, the potential-dependent impedance of the NMC cathode was measured in the three-electrode PAT cells by charging the NMC cathode at C/20 to various potentials vs. the lithium reference electrode: 3.8, 4.1, 4.3, 4.5, and 4.6 V for NMC111 and NMC811 and also 4.7, 4.8, and 4.9 V for NMC111. The cell was held at each potential for 1 h to reach a steady-state and then allowed to rest at open circuit potential (OCP) for 1 h before the EIS was measured. Potential-controlled EIS was conducted in a frequency range of 500 kHz to 10 mHz with an AC voltage perturbation of 5 mV (Biologic VMP3). The SOC of NMC at each potential was calculated based on the charge passed and a theoretical capacity of 277.9 mAh g^–1^ for NMC111 and 275.5 mAh g^–1^ for NMC811. All electrochemical protocols were performed in climate chambers set at 25 °C. Two or more cells were evaluated for each condition to ensure reproducibility, which is indicated by error bars in the respective figures.

### Online Electrochemical Mass Spectrometry (OEMS)

The OEMS system consists of a stainless-steel tube carrying gas through the electrochemical cell (Swagelok type), connected through self-sealing quick-connects (Beswick Engineering) so the system is never exposed to air, to a mass spectrometer. Mixtures of several gases can be selected via mass flow controllers (Bronkhorst), with a pressure controller (Bronkhorst) maintaining the gas line at a constant 1.1 bar(a). For this work, Ar (BOC N6.0), connected to a purifier (Bronkhorst) before flow control, was used as the carrier gas. The gas line is connected to a quadrupole mass spectrometer (Pfeiffer) through a heated capillary (120 °C) to prevent condensation. A potentiostat (Ivium) controls the electrochemical operations.

Calibration was performed by flowing through the system a mixture of H_2_, CO, C_2_H_4_, O_2_, and CO_2_ (1000 ppm each) in Ar (BOC N6.0) to establish a correlation between channel ion currents and gas molar flow. The molar flow of CO was established by subtracting the proportional amount of CO_2_ measured in channel *m*/*z* = 44 to channel *m*/*z* = 28 according to the relative intensities of both channels in the spectral pattern of CO_2_.

Cells for OEMS were assembled in half cell setup with an 18 mm diameter cathode, 15.6 mm diameter metallic Li chip counter/reference electrode (0.25 mm thickness, LTS Research Laboratories), and 25 mm diameter glass fiber separator (Whatman, GF/B) soaked in 300 μL of electrolyte. In some experiments, a 25 mm diameter lithium ion conducting glass-ceramic (LICGC) separator (0.15 mm thickness, Ohara AG-01) was placed between two 22 mm diameter glass fiber separators to prevent the migration of decomposition species in the electrolyte to the opposite electrode. After assembly, the cells were connected to the OEMS system and the potentiostat and held at the OCP for 6 h before starting the electrochemical protocol. The cells were cycled using an analogous protocol to that described for the coin cells above. Specifically, the cells were charged galvanostatically at C/20 to 4.6 V vs Li/Li^+^ and held at that potential for 40 h, after which they were discharged at C/20 to 2.5 V vs Li/Li^+^ and held at OCP for at least 12 h.

### Materials Characterization

The surface area of the NMC powders was determined by nitrogen gas physisorption at 77 K, measuring isothermally at 10 points between 0.07 ≤ *p*/*p*_0_ ≤ 0.30 (3Flex, Micromeritics). The water content of the electrolytes was measured by Karl Fischer titration (899 Coulometer, Metrohm). NMR experiments were conducted on a Bruker Avance III HD spectrometer equipped with a 11.7 T magnet (ν_1H_ = 500 MHz) using a BBO probe. ^1^H, ^19^F, and ^31^P NMR spectra were acquired using a one pulse pulse sequence; ^19^F and ^31^P experiments were conducted without the use of ^1^H decoupling. ^1^H chemical shifts were referenced to the DMSO-*d*_6_ solvent peak at 2.50 ppm. ^19^F and ^31^P chemical shifts were referenced to LiPF_6_ at −74.5 and −145.0 ppm, respectively. Pristine electrolyte was measured by pipetting 40 μL of the electrolyte solution into 0.7 mL of DMSO-*d*_6_ (99.9 atom % D, 99% CP, Sigma-Aldrich), which was transferred to an airtight NMR tube fitted with a Young’s tap.

After the 60 h VH protocol, the NMC/LTO coin cells were disassembled in an Ar filled glovebox. The separator was extracted and soaked in 0.7 mL of DMSO-*d*_6_ for 5 min. The solution was transferred to an airtight NMR tube fitted with a Young’s tap for measurement. The NMC and LTO electrodes were extracted, rinsed with 1 mL of dimethyl carbonate (DMC, anhydrous, ≥99%, Sigma-Aldrich), and vacuum-dried at ambient temperature for 1 h prior to measurement by XPS and HRTEM.

XPS measurements were performed using a Thermo Scientific Nexsa X-ray photoelectron spectrometer system utilizing Al Kα X-rays. The electrodes were transferred inertly into the system without air exposure. Energy calibration was performed by setting the carbon black feature in the C 1s core level region to 284.4 eV for the NMC electrodes and the Ti 2p_3/2_ peak to 459.3 eV for the LTO electrodes. A Shirley type background was subtracted from all spectra besides the region containing the transition metal 3p core levels. The probing depth corresponding to the intensity of 95% of the emitted photoelectrons was calculated according to^27^
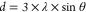
where *d* is the probing depth, λ is the inelastic mean free path, and θ is the electron take off angle, i.e., the angle between the sample surface and the analyzer. The inelastic mean free path was calculated to 3.38 nm based on the photoelectrons emitted from the Ti 2p orbital traveling through a SEI consisting of polyethylene and 3.19 nm for photoelectrons emitted from the O 1s orbital traveling through a CEI consisting of polyethylene.^28^ The equation assumes the surface is flat and the chemical composition is known and homogeneous. This is not the case for the electrodes in this study, although it should still serve as a reasonable approximation.

The NMC cathode materials (including NMC111 or NMC811 particles, conductive carbon, and PVDF) were scratched from the NMC electrode on an aluminum current collector and ground with an agate pestle and mortar. The powder material was then transferred onto the holey carbon copper grid in an Ar filled glovebox. A single tilt holder was used for TEM (JEM-2100Plus, JEOL) characterization.

Elemental analysis was performed using inductively coupled plasma-optical emission spectroscopy (ICP-OES, Thermoscientific) calibrated with standards prepared from an ICP multielement solution (VWR, Aristar). For ICP-OES analysis, NMC/LTO coin cells were constructed with 120 μL of electrolyte and either three separators (Celgard, glass fiber, Celgard; for LP57 and EMC electrolyte) or one separator (glass fiber; for EC electrolyte). The higher volume of electrolyte (transferred within the glass fiber separator) enables the extraction of a sufficient volume for analysis, while the Celgard separator facilitates easier separation of the electrodes from the glass fiber. After cycling with the 60 h VH protocol, the cells were disassembled in an Ar filled glovebox. The LTO anode and separators were extracted, placed in a 15 mL polypropylene tube and centrifuged at 5000 rpm for 10 min. The LTO anode and the separators were removed and 4.0 mL of ∼2% nitric acid (diluted from concentrated nitric acid; 67–69%, trace metal grade, Fischer Chemical) was added to the extracted electrolyte (34–70 μL) before analysis. The LTO coating was scraped from the current collector (19–26 mg_LTO_) and soaked in 248 μL of 5.1 M nitric acid for 5 days before being diluted to 4.0 mL with 3.75 mL of water for analysis. The LTO coating scraped from uncycled electrodes was measured as a baseline.

## Results

### Electrochemistry

**Figure 1:**
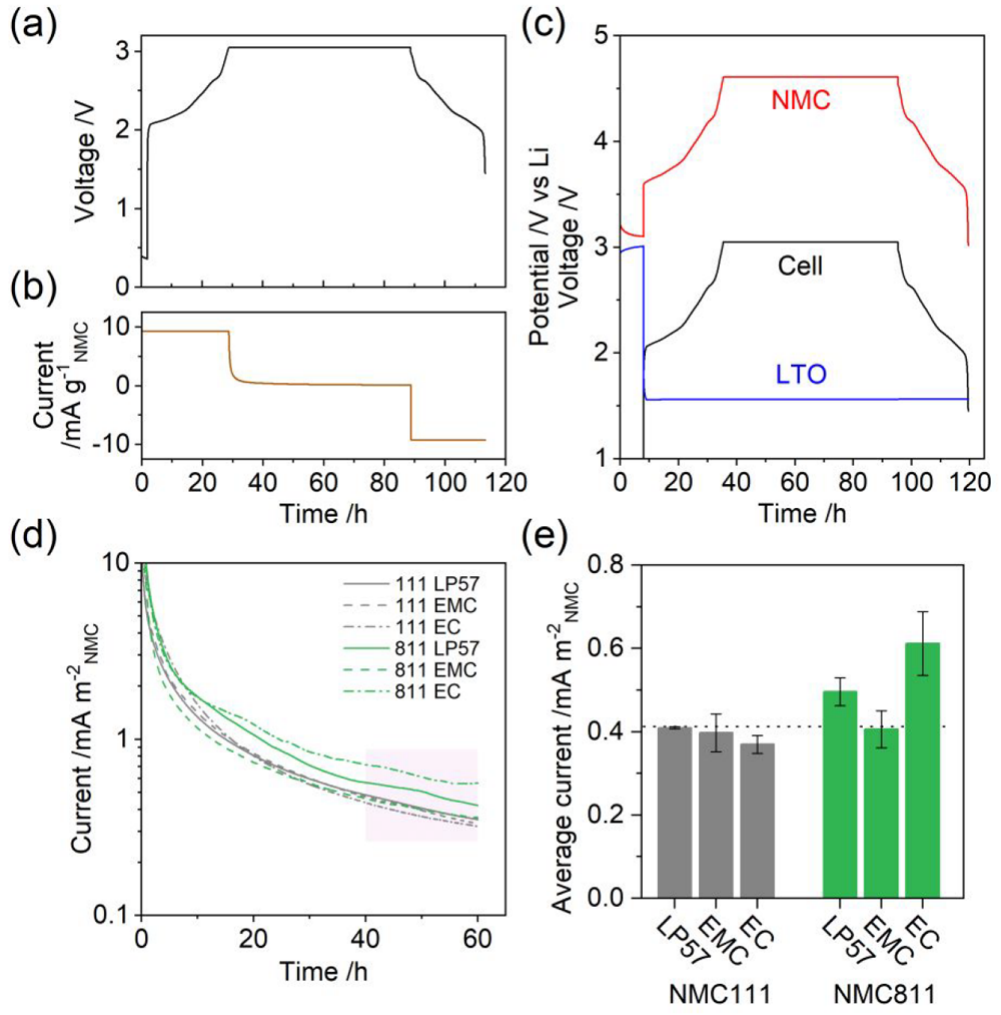
Representative (a) voltage and (b) current profiles for a NMC/LTO coin cell during the first charge–discharge cycle between 1.45 and 3.05 V at C/20 with a 60 h potentiostatic hold at 3.05 V. Data is shown for NMC811/LTO with LP57 electrolyte. (c) Potential profiles of the NMC811 cathode and LTO anode in a three-electrode cell with a Li metal reference electrode. (d) Oxidation current during the potentiostatic hold and (e) the average current in the final 20 h of the potentiostatic hold for both NMC111 and 811 with electrolytes LP57, 1.5 M LiPF_6_ in ethyl methyl carbonate (EMC), and 1.5 M LiPF_6_ in ethylene carbonate (EC). The current in parts d and e is normalized by the NMC BET surface area. The error bars in part e represent the spread obtained from two or more duplicate cells.

Figure 1a,b shows the voltage profile and current trace for an NMC811/LTO cell with LP57 electrolyte (1 M LiPF_6_ in EC/EMC 3/7 by volume) during the first charge–discharge cycle with a 60 h potentiostatic hold at 3.05 V, referred to henceforth as the “60 h voltage hold (VH) protocol”. The three-electrode cell measurement in Figure 1c illustrates that LTO exhibits a potential plateau at 1.55 V vs Li/Li^+^ during charging (lithiation), and therefore at a full cell voltage of 3.05 V, the NMC potential is 4.6 V vs Li/Li^+^.

Figure 1d shows the oxidation current (normalized to the NMC BET surface area, Table S1) during the potentiostatic hold with NMC111 and 811 and for three electrolyte solutions. A conventional LP57 electrolyte is tested alongside two model, single solvent electrolytes, 1.5 M LiPF_6_ in EMC and 1.5 M LiPF_6_ in EC. Potential profiles for all the conditions tested are shown in Figure S1. Owing to the lower delithiation potential of the Ni-rich NMC811 compared to NMC111, the amount of capacity extracted in the charge to 3.05 V, and hence the state-of-charge (SOC), is greater for NMC811 (90% SOC) than for NMC111 (76% SOC), as shown in Figure S2. A 1.5 M salt concentration was used for the EMC electrolyte to improve the ionic conductivity^2^ and for the EC electrolyte to lower the viscosity,^29^ and glass microfiber separators (grade GF/A) were used in cells with the EC electrolyte since it does not wet Celgard 2325 polymer separator. Control experiments for the influence of the salt concentration (1.0 and 1.5 M) and separator (Celgard and GF/A) on the current in the potentiostatic hold are provided in Figure S3 and show equivalent electrochemical behavior. The water contents in the three electrolytes as measured by Karl Fischer titration are given in Table S2.

In the first 20–30 h of the potentiostatic hold, the current decays rapidly as the electrolyte polarization relaxes and as the concentration of lithium in the bulk of the NMC particles reach the equilibrium value set by the applied potential. At later times, the current is dominated by oxidative decomposition reactions at the electrolyte–NMC interface. To quantitatively compare the stability of each electrolyte at the NMC interface, the average current in the final 20 h of the potentiostatic hold is plotted in Figure 1e. For NMC111, the average current is largely independent of the electrolyte, at a value of 0.40(3) mA m^–2^_NMC_. With LP57, NMC811 results in a 21% higher average current (0.50(3) mA m^–2^_NMC_), in line with recent literature reporting poorer cathode surface/oxygen stability for Ni-rich NMCs at an equivalent cathode potential.^4,30^ Further, for NMC811, the current is dependent on the electrolyte solvent(s) and is 18% lower for EMC electrolyte and 23% higher for EC electrolyte compared to LP57. As mentioned earlier, the current measured can have contributions from lattice oxygen release and electrolyte oxidation, which releases gases and produces soluble and insoluble electrolyte degradation products. The gas, liquid, and solid phases resulting from these processes are next characterized in situ by OEMS and ex situ by solution-state NMR on extracted electrolyte and XPS on the extracted electrodes. TM dissolution from NMC is also investigated by ICP-OES on the extracted electrolyte and anodes.

### Gas Evolution: Online Electrochemical Mass Spectroscopy (OEMS)

**Figure 2:**
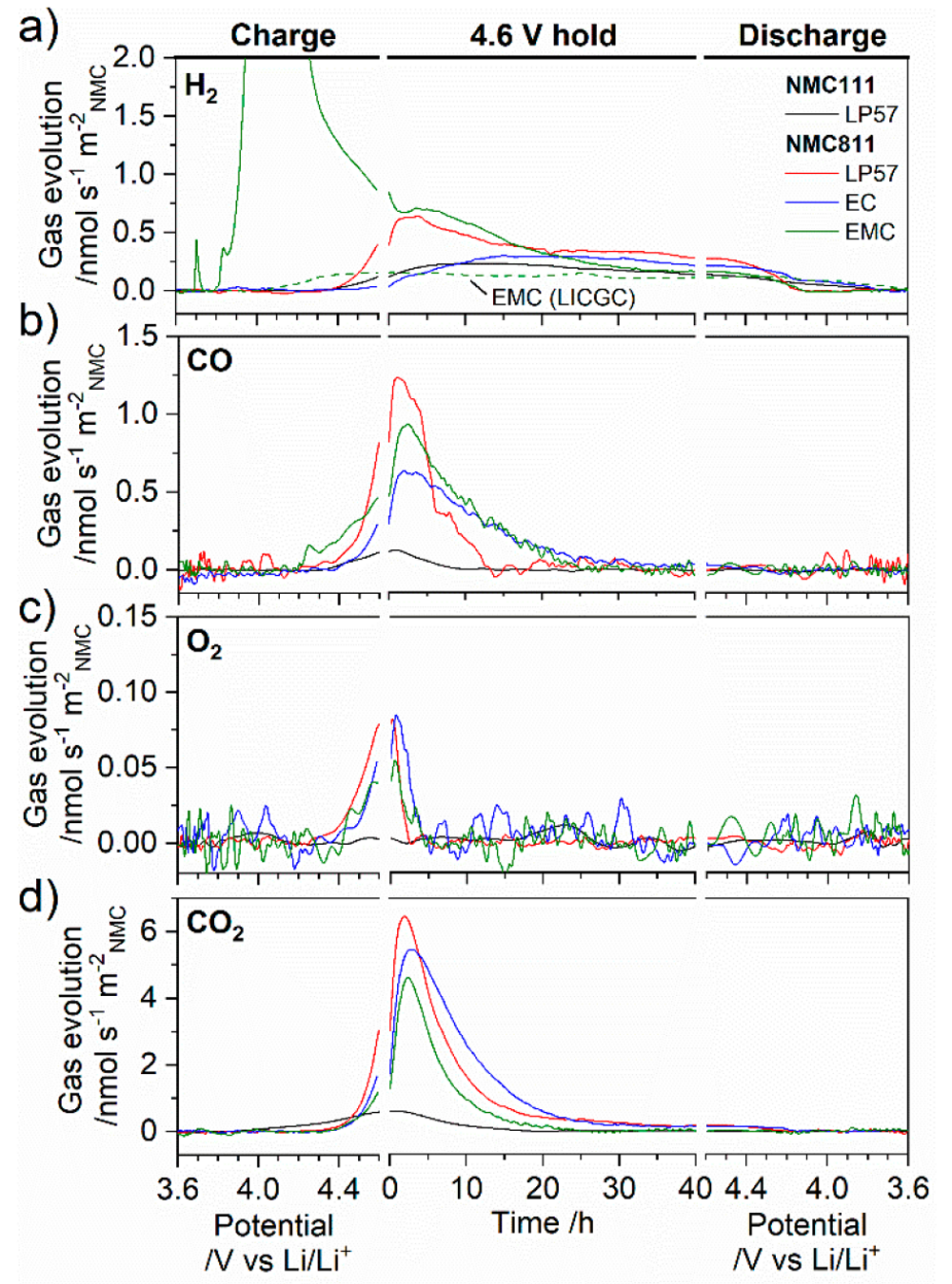
Evolution of (a) H_2_, (b) CO, (c) O_2_, and (d) CO_2_ as determined from the OEMS channels *m*/*z* = 2, 28, 32, and 44, respectively, and normalized to the NMC surface area: for NMC/Li cells during the first charge–discharge cycle between 2.5 and 4.6 V at C/20 with a 40 h potentiostatic hold at 4.6 V and for NMC111 with LP57 electrolyte and NMC811 with electrolytes LP57, 1.5 M LiPF_6_ in ethyl methyl carbonate (EMC), and 1.5 M LiPF_6_ in ethylene carbonate (EC). Data are plotted as a function of potential for the charge and discharge and time for the potentiostatic hold. The evolution of H_2_ for a NMC811/Li cell with a lithium ion conducting glass-ceramic separator (Ohara, LICGC) with 1.5 M LiPF_6_ in EMC as the catholyte and LP57 as the anolyte is also shown in part a as a dashed green line.

For reasons explained in Supplementary note S1, an NMC/Li half-cell is used for the OEMS experiments. In brief, decoupling the cathode and anode gas evolution from NMC/Li cells is more straightforward than from NMC/LTO cells; a lithium ion conducting glass-ceramic separator (Ohara, LICGC) is used for some experiments to decouple the anode from cathode processes. The current rate and potential applied to the NMC cathode (shown in Figure S4 for all the conditions tested) were kept the same as in Figure 1, although the potentiostatic hold time was reduced to 40 h since most of the gas evolution took place at times <40 h. Gas evolution profiles are shown in Figure 2 as a function of potential for charge and discharge and time for the potentiostatic hold. For NMC111 with LP57 electrolyte, the onset for CO_2_ evolution is ∼3.8 V vs Li/Li^+^. This is characteristic of the oxidation of carbonate impurities^31−33^ that form on the surface of NMC particles during long-term storage (even in a dry room environment) via reactions with carbon dioxide and water.^34,35^ These surface contaminants can (largely) be removed via thermal treatment,^36^ and additional OEMS experiments conducted on electrodes prepared with annealed NMC111 powder (750 °C for 4 h in air) yield a CO_2_ onset potential of ∼4.4–4.5 V vs Li/Li^+^ (Figure S5). While the NMC111 cathodes appear to have “aged”, NMC811 does not evolve CO_2_ at potentials below 4.4 V vs Li/Li^+^ indicating that the NMC811 sample is largely free of surface impurities. It is well understood that the high surface reactivity of Ni-rich cathodes make them more prone to the formation of surface impurities during storage,^37,38^ and hence it is likely that the storage conditions and/or time are different for the NMC111 and 811 electrodes used in this work.

**Figure 3:**
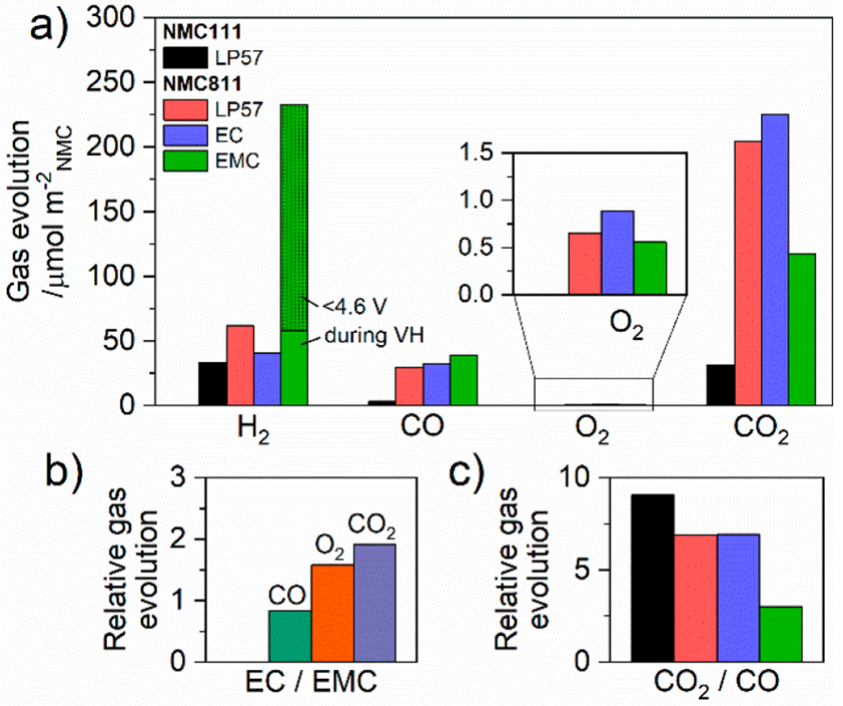
(a) Quantity of H_2_, CO, O_2_, and CO_2_ gases evolved in the OEMS experiments shown in Figure 2. The inset shows a magnified view of O_2_. (b) Fraction of CO, O_2_, and CO_2_ evolved with NMC811 and 1.5 M LiPF_6_ in ethylene carbonate (EC) electrolyte relative to 1.5 M LiPF_6_ in ethyl methyl carbonate (EMC) electrolyte (EC/EMC). (c) Fraction of CO_2_ relative to CO (CO_2_/CO) for each NMC-electrolyte pairing.

At ∼4.4 V vs Li/Li^+^, and independent of the electrolyte, the signals from CO, O_2_, and CO_2_ begin to rise simultaneously for NMC811 (Figure 2). Gasteiger and co-workers also observed this phenomenon^6,10^ and have shown that reactive lattice oxygen (e.g., singlet oxygen) released from NMC reacts with the electrolyte solvent producing CO and CO_2_.^11,12^ O_2_ detected in the OEMS experiment may arise from the ground state (i.e., triplet) O_2_ release from the lattice and/or from deactivation of ROS via nonradiative (electronic-to-vibrational coupling to solvent molecules) and radiative transition to the ground state.^12^ The signals from CO, O_2_, and CO_2_ continue to rise before reaching a maximum at, or shortly after (<3 h), the start of the potentiostatic hold. For LP57, the total quantity of each gas released, shown in Figure 3a, is dependent on the NMC composition: the amount of CO and CO_2_ evolved are 8.5 and 6.4 times higher for NMC811 (29.7 and 204 μmol m^–2^_NMC_, respectively) compared to NMC111 (3.5 and 31.7 μmol m^–2^_NMC_, respectively), with the amount of CO_2_ for NMC111 over-represented due to CO_2_ evolution from the surface impurities present. Note that differences in the slurry preparation and electrode manufacture limit direct comparison between the annealed NMC111 data in Figure S5 and the other conditions in Figure 2. Further, O_2_ evolution is not detected from NMC111 (Figure 2c and Figure S5) but is clearly evident for NMC811 (0.7 μmol m^–2^_NMC_), which is likely related to the different NMC SOC at 4.6 V vs Li/Li^+^ (i.e., 90% for NMC811 vs 76% for NMC111, Figure S2). However, the onset of CO_2_ release at ∼4.4–4.5 V vs Li/Li^+^ for annealed NMC111 is likely indirect evidence of a small amount of lattice oxygen release. This is consistent with a ∼ 4.6 V vs Li/Li^+^ onset potential for O_2_ evolution from NMC111 reported previously.^10^ It should be noted that purely electrochemical oxidation of electrolyte solvents also produces CO_2_ and CO gas^10,39,40^ (oxidation onset potentials in the range 4.5–6.0 V vs Li/Li^+^ have been reported^41−46^) and therefore may contribute to the gas evolution in Figure 2 and Figure S5. Excluding any catalytic effect of Ni (ruled out in experiments reported by Jung et al.^6,10^), the gas evolution with NMC111 provides an upper limit for the contribution from direct electrochemical oxidation. Therefore, the majority of the CO_2_ and CO evolution with NMC811 stems from chemical oxidation pathways or a coupled chemical and electrochemical process. Even if we assume no lattice oxygen release from NMC111 at 4.6 V vs Li/Li^+^, we estimate that electrochemical oxidation can account for at most ∼12% of the evolved CO_2_ for NMC811, in line with calculations by Jung et al.^6^

Figures 2 and 3a also show that the gas evolution with NMC811 is dependent on the electrolyte. Compared to NMC111, enhanced CO and CO_2_ gas evolution is observed for NMC811 with LP57, EC, and EMC only electrolytes, indicating that reactive lattice oxygen reacts with both cyclic and linear carbonates, although the quantity and ratio of gases vary. The ratio of each gas evolved with EC relative to EMC electrolyte (EC/EMC, Figure 3b) is particularly insightful as it probes the relative gassing behavior of the individual solvents. With EC electrolyte, the amount of CO_2_ evolved (225 μmol m^–2^_NMC_) is 1.9 times higher than with EMC electrolyte (117 μmol m^–2^_NMC_), while LP57 (204 μmol m^–2^_NMC_) shows only a slight reduction compared to EC electrolyte. A similar trend is observed for O_2_, with 1.6 times more O_2_ with EC electrolyte (0.886 μmol m^–2^_NMC_) relative to EMC electrolyte (0.560 μmol m^–2^_NMC_), although the quantities of O_2_ are more than 10^2^ times lower than CO_2_ and therefore more susceptible to errors. Conversely, the amount of CO evolved is marginally higher for EMC electrolyte (38.7 μmol m^–2^_NMC_) compared to that for EC electrolyte (32.5 μmol m^–2^_NMC_) and LP57 (29.7 μmol m^–2^_NMC_), yielding an EC/EMC fraction of 0.8. The similar quantity of each gas evolved with LP57 and EC electrolyte, along with the equivalent CO_2_/CO fraction of 6.9 (compared to 3.0 for EMC electrolyte, Figure 3c) strongly suggests that the NMC-induced gassing in LP57 is dominated by EC.

**Scheme 1:**
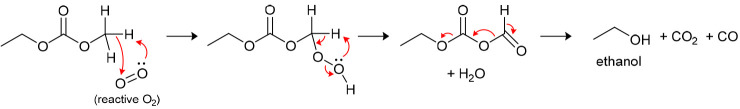
Proposed Mechanism for the Chemical Oxidation of Ethyl Methyl Carbonate (EMC), Showing the Generation of CO_2_, CO, and H_2_O

Taking an O_2_/CO_2_ mole ratio of 1:1 for the reaction of EC and EMC with reactive oxygen (as proposed by Jung et al. for EC,^6^ and as proposed for EMC in the Discussion section (see Scheme 1), the higher fraction of CO_2_ evolved in the EC electrolyte (1.9 times) indicates significantly more lattice oxygen release from NMC811 with EC-containing electrolytes. This finding is also supported by the proposed O_2_/CO mole ratios and the relative quantities of CO produced, which will be discussed in further detail in the Discussion section.

Turning now to the H_2_ evolution in Figure 2a, for the EC-containing electrolytes, there is a clear onset potential between 4.4 and 4.6 V vs Li/Li^+^, similar to that seen for CO, O_2_, and CO_2_. H_2_ is then evolved throughout the potentiostatic hold and only stops once the NMC potential drops below ∼4.2 V vs Li/Li^+^ on discharge. Reduction of trace water in the electrolyte at the anode (Table S3) cannot account for this trend since (i) with a lithium metal anode this process would be potential independent, and (ii) the total quantity of H_2_ evolved in each electrolyte is >30 times that expected from the measured trace water content alone; see the calculations in Supplementary note S2. Instead, the observed H_2_ evolution is likely the result of electrode crosstalk, in which protic oxidation species formed at high SOCs at the cathode diffuse to the anode where they are reduced.^40^ The identity of these protic species could include water, protons, and protic electrolyte solvent oxidation fragments.^6,47,48^ Mechanisms for the formation of these species are discussed further in the Discussion section. The sustained evolution of H_2_ throughout the potentiostatic hold indicates that protic species are continuously produced when the NMC is above the threshold potential.

Unfortunately, the relative quantity of H_2_ evolved with different electrolytes is not necessarily an accurate measure of the amount of protic species formed at the cathode. A more effective SEI on the anode, such as that formed with EC-containing electrolytes or other specialized additives, hinders the reduction of protic species^40^ which may then react via alternate pathways. This likely explains the much larger H_2_ evolution with EMC electrolyte (Figure 2a, 3.8 times that with LP57), since EMC is a poor SEI former on lithium metal and graphite anodes, and in the latter case forming an SEI that is nonuniform and thinner compared to an EC-based SEI.^49^ To prove this hypothesis, a NMC811/Li cell was built with a lithium ion conducting glass-ceramic separator to block the liquid-state diffusion of protic species to the anode. EMC electrolyte was used as the catholyte and LP57 as the anolyte. Schematics of the cell stack with and without the Ohara glass separator are shown in Figure S6, and the potential profiles are shown in Figure S4b. As expected, the total amount of CO_2_ evolved is very similar between the two runs with EMC electrolyte (117 μmol m^–2^_NMC_ and 108 μmol m^–2^_NMC_ with and without the Ohara glass separator, respectively), which validates the comparison. As shown in Figure 2a, with the glass-ceramic separator and LP57 anolyte, the quantity of H_2_ detected is significantly lower, although the detection of a small amount of H_2_ suggests an imperfect seal between the two compartments of the cell. Finally, we note that variability in the treatment of the lithium metal anode (e.g., scraping) prior to the experiment may also introduce variability in the active surface area and hence the rate of H_2_ evolution between experiments with the same electrolyte.

### NMC Impedance: Three-Electrode Electrochemical Impedance Spectroscopy (EIS)

**Figure 4:**
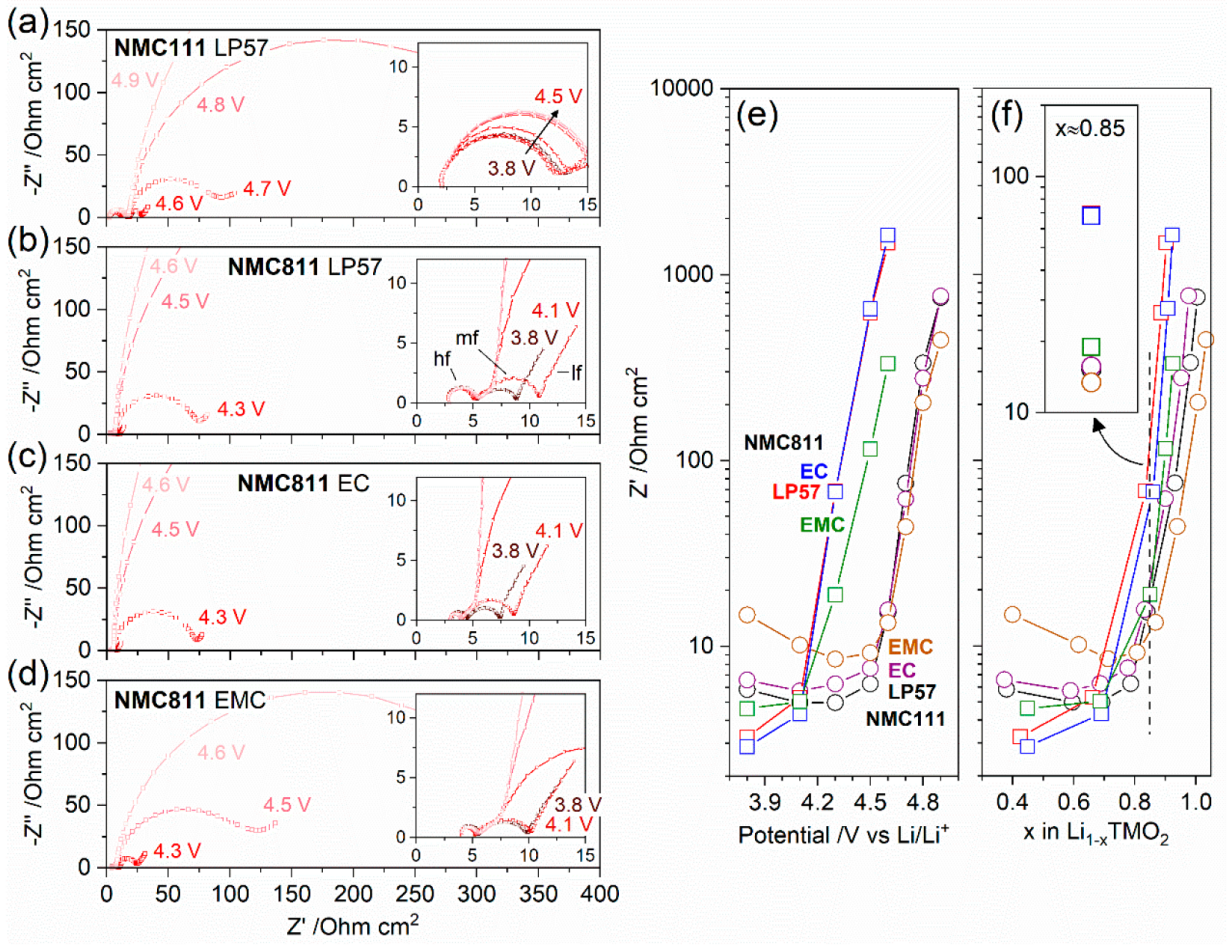
(a–d) Nyquist impedance plots of the NMC cathode as a function of potential measured in a three-electrode NMC/LTO cell with a Li metal reference electrode after the first charge–discharge cycle between 2.5 and 4.6 V at C/20 with a 60 h potentiostatic hold at 4.6 V for (a) NMC111 and (b–d) NMC811 with electrolytes: (a,b) LP57, (c) 1.5 M LiPF_6_ in ethylene carbonate (EC), and (d) 1.5 M LiPF_6_ in ethyl methyl carbonate (EMC). Electrolyte-oxide interfacial impedance, plotted on a logarithmic scale, as a function of NMC (e) potential and (f) state of charge (SOC, i.e., *x* in Li_1–*x*_TMO_2_) for NMC111 (circles) and NMC811 (squares). The inset in part f highlights the data at SOC value *x* ≈ 0.85.

The potential/SOC-dependent impedance of NMC after the 60 h VH protocol was measured in three-electrode format, controlling the potential of the NMC versus the lithium metal reference electrode. The electrochemical protocols applied for NMC111 and 811 are shown in Figure S7. Figure 4a–d shows the Nyquist plots for NMC111 with LP57 (NMC111 with EC and EMC electrolytes are shown in Figure S8) and for NMC811 with LP57, EC, and EMC electrolytes—the NMC potential for each spectrum is indicated in Figure 4a–d and the corresponding SOC is provided in Table S4. Three features are evident (labeled in the inset in Figure 4b), a high frequency semicircle (hf), a midfrequency semicircle (mf), and a Warburg impedance tail at low frequencies (lf). The diameter of the midfrequency semicircle, which can be attributed to the electrolyte-oxide interfacial impedance,^8,50^ was extracted by fitting a simplified equivalent circuit to the data (see Supplementary note S3) and is plotted as a function of potential and SOC in Figure 4e,f. The interfacial resistance shows a huge growth at potentials >4.1 V vs Li/Li^+^ for NMC811 and >4.5 V vs Li/Li^+^ for NMC111 (Figure 4e). This apparent difference is largely due to NMC811 approaching the delithiated state at lower potentials than NMC111, which is accounted for in Figure 4f, where the resistance is plotted as a function of Li content. For fully delithiated NMC a quasi-infinite charge transfer resistance is expected, referred to as a blocking condition,^51^ justifying the general trend seen in the data. Nevertheless, at high SOCs (*x* > 0.7 in Li_1–*x*_TMO_2_), the impedance is dependent on both the NMC composition and the electrolyte. With LP57, the impedance at *x* > 0.7 for NMC811 is higher than NMC111, e.g., at *x* = 0.84(1) the impedance of NMC811 (68.8 Ω.cm^2^) is 4.5 times that of NMC111 (15.2 Ω cm^2^), see the inset in Figure 4f. In terms of the electrolyte dependence, for *x* > 0.7, NMC811 with LP57 and EC electrolyte exhibit similar impedance values, while with EMC electrolyte the impedance is 70–80% lower, e.g., at *x* = 0.85(1) the impedance with EC electrolyte (67.8 Ω.cm^2^) is 3.6 times that of EMC electrolyte (18.9 Ω cm^2^) (inset in Figure 4f). This indicates that the impedance growth in LP57 is dominated by the EC solvent contribution. The EC in LP57 was also found to dominate the gassing behavior with NMC811, which stems from lattice O_2_ release (see above). This suggests a correlation between impedance and gas evolution, which will be explored in the Discussion section.

### NMC Surface Reconstruction: High-Resolution TEM

**Figure 5:**
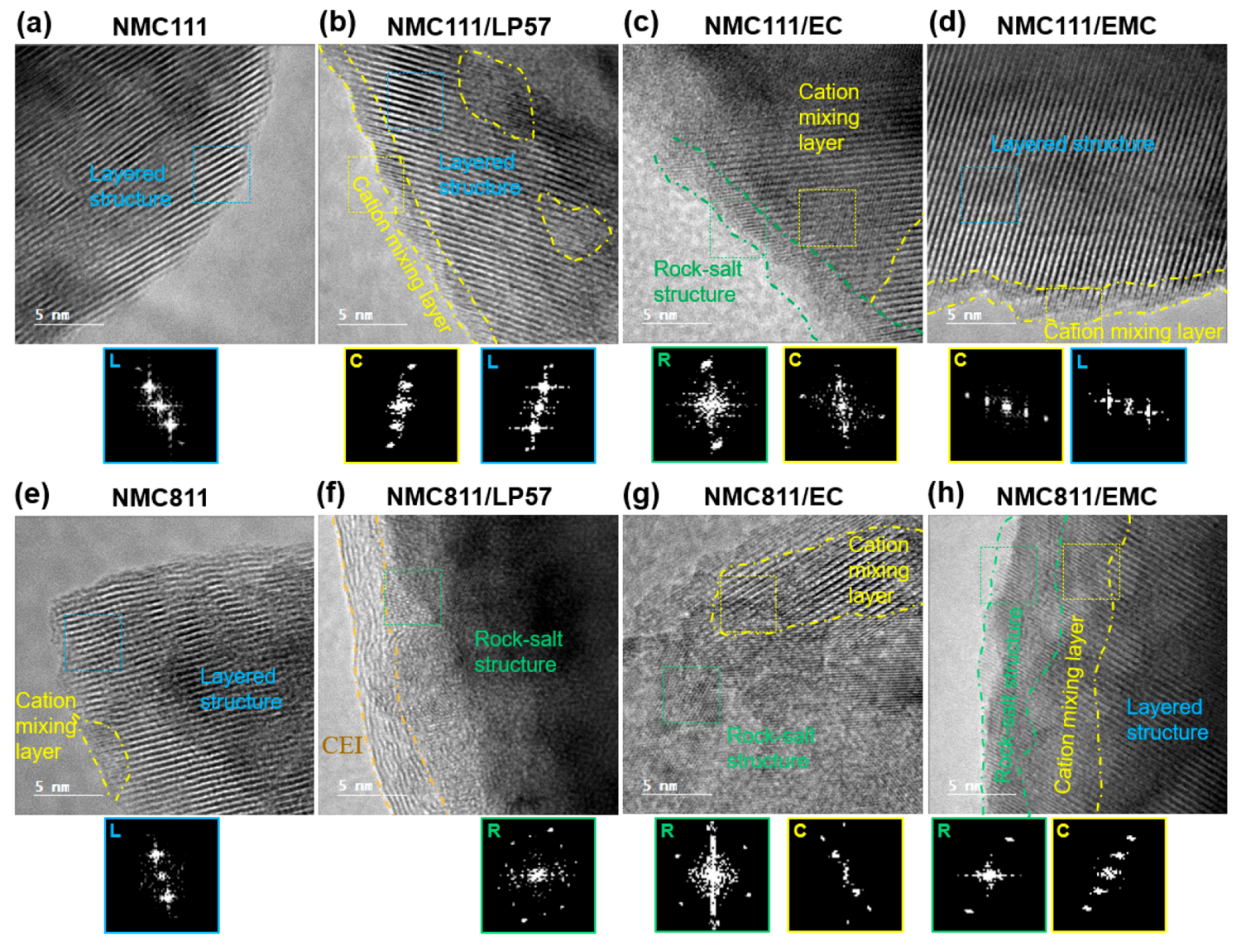
High-resolution TEM images and corresponding fast Fourier transformation (FFT) images of NMC111 (a–d) and NMC811 (e–h) in the pristine state (a,e) and after the first charge–discharge cycle between 2.5 and 4.6 V at C/20 with a 60 h potentiostatic hold at 4.6 V with electrolytes LP57 (b,f), 1.5 M LiPF_6_ in ethylene carbonate (EC) (c,g), and 1.5 M LiPF_6_ in ethyl methyl carbonate (EMC) (d,h). The dashed squares indicate where the FFTs are analyzed. The letters L, R, and C in the FFTs stand for layered structure, rock-salt structure, and cation mixing layer, respectively.

TEM was used to study the interfacial structure of NMC nanoparticles in the pristine state and after the 60 h VH protocol. The HRTEM images in Figure 5a,e show the layered structure of pristine NMC111 and NMC811, respectively, with the layered structure being confirmed by the corresponding fast Fourier transformation (FFT) images. Pristine NMC811 also has some layers with cation mixing between the Li and TM layers, as shown in Figure 5e, likely stemming from the higher fraction of Ni^2+^ and the propensity for Li/Ni site-disorder; dislocations/grain boundaries are also seen. Formation of a surface reconstruction layer (SRL) was observed on the surface of NMC111 (Figure 5b–d) and NMC811 (Figure 5f–h) after the 60 h VH protocol with LP57, EC electrolyte, and EMC electrolyte. The EC electrolyte shows a thicker SRL on the surface of NMC111 than EMC electrolyte or LP57, including rock-salt structure and cation mixing layer, as shown in Figure 5c and the corresponding FFTs. In comparison, there are only fine SRL structures, mainly a cation mixing layer of 2–4 nm in thickness, on the surface of the NMC111 for LP57 and EMC electrolyte, as shown in Figure 5b,d and their corresponding FFTs, respectively. However, some regions with cation mixing can be seen in the bulk area of NMC111 with LP57, as shown in Figure 5b.

Compared with NMC111, more and thicker SRLs form on the surface of NMC811 after the 60 h VH protocol with LP57, EC electrolyte, and EMC electrolyte, as shown in Figure 5f–h, respectively. LP57 electrolyte leads to the formation of a thick rock-salt structure on the surface of NMC811, as shown in Figure 5f and the corresponding FFT. Figure 5f also shows the probable formation of cathode electrolyte interface (CEI) on the surface of NMC811 in LP57 solution. A thick rock-salt structure can also be seen on the surface of NMC811 with the EC electrolyte, as shown in Figure 5g and the corresponding FFTs, and some parts of the particle have a cation mixing layer. In contrast, EMC electrolyte leads to a uniform rock-salt structure and cation mixing layer with a thickness of 3–5 nm on the surface of NMC811, as shown in Figure 5h and the corresponding FFTs. While we recognize that only a minute fraction of the electrode material is sampled by HRTEM, the results shown are representative of many particles sampled at random from the electrode. Moreover, they are in accord with the electrochemistry and gas analysis studies described above.

### Insoluble Electrolyte Degradation: XPS

**Figure 6:**
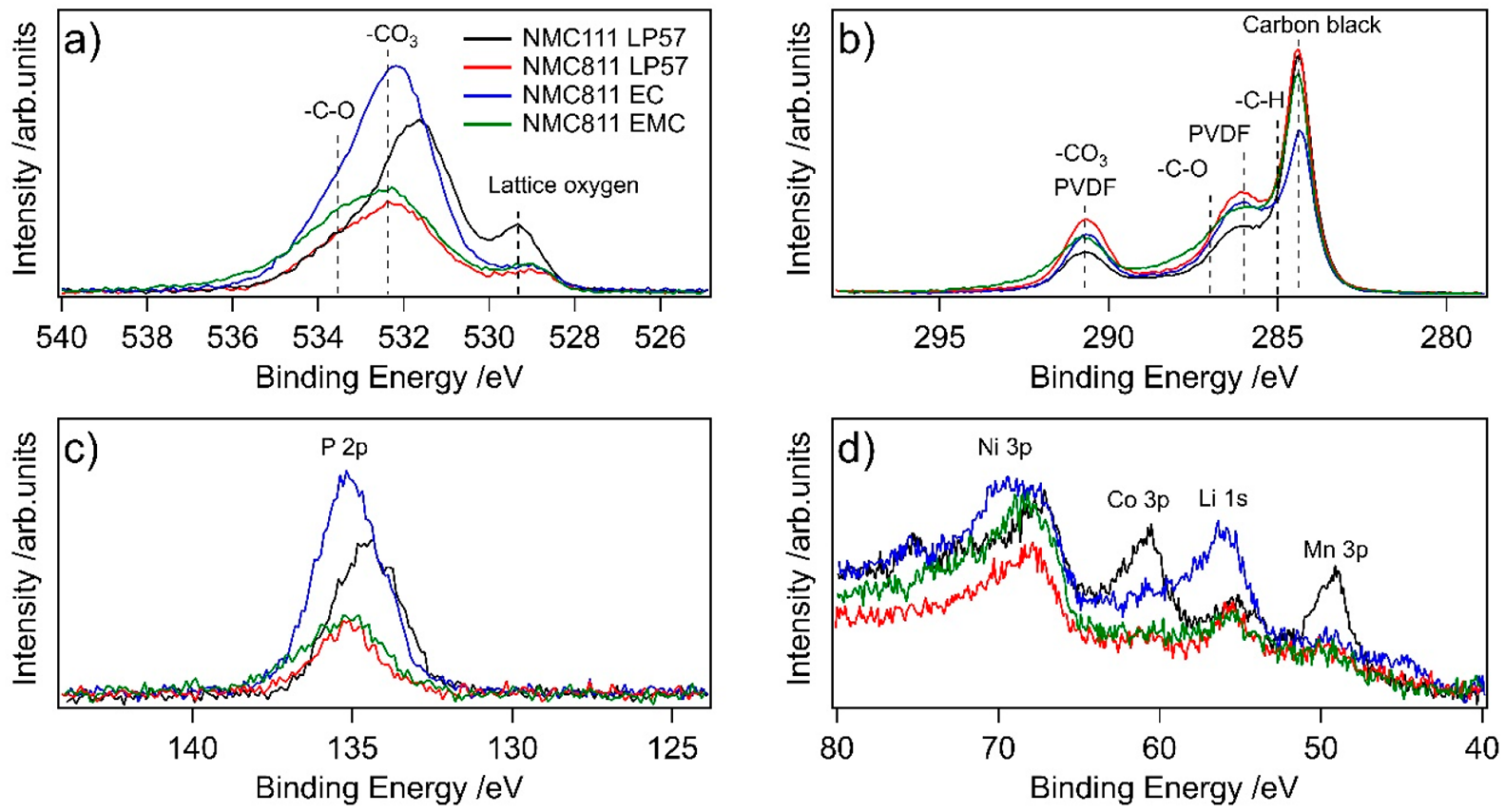
XPS spectra of NMC electrodes extracted from NMC/LTO cells after the first charge–discharge cycle between 1.45 and 3.05 V at C/20 with a 60 h potentiostatic hold at 3.05 V for NMC111 and 811 with electrolytes LP57, 1.5 M LiPF_6_ in ethyl methyl carbonate (EMC), and 1.5 M LiPF_6_ in ethylene carbonate (EC): (a) O 1s spectra, (b) C 1s spectra, (c) P 2p spectra, and (d) Ni 3p, Co 3p, Li 1s, and Mn 3p core levels plotted without background subtraction.

Figure 6 shows the XPS spectra of the NMC electrodes after the 60 h VH protocol using different electrolytes. Comparing NMC111 and 811 with LP57, the O 1s spectra in Figure 6a show that the lattice oxygen peak (∼529.4 eV) is much higher in intensity for the NMC111 electrode compared to the NMC811 electrode, indicating a thinner CEI is formed for NMC111. In the corresponding P 2p spectra shown in Figure 6c, a peak is seen at 135 eV, likely coming from degradation of the LiPF_6_ salt into Li_*x*_PO_*y*_F_*z*_ compounds, which is more intense for NMC111 than 811 in LP57. Given the thinner CEI of the NMC111, it may be that the phosphorus species are less buried beneath the organic CEI components and/or that the average phosphorus content of the CEI is increased. In either case, the larger phosphorus signal close to the CEI surface is consistent with the hypothesis that the degraded salt stabilizes the electrode preventing further degradation from organic compounds. In the region containing the transition metal 3p core levels, peaks corresponding to cobalt (∼61 eV) and manganese (∼49 eV) are clearly apparent for NMC111 in contrast to 811 where these are barely detectable above the background, consistent with the lower fractions of Mn and Co in NMC811. The same trends observed in Figure 6 for LP57 (i.e., a thinner CEI yet more phosphorus species for NMC111) are also observed for EC electrolyte in Figure S10.

Comparing the role of the electrolyte on the Ni-rich NMC surface, the lattice oxygen peaks for all NMC811 electrodes have a similar intensity suggesting no major difference in the CEI layer thickness, although the LP57 electrolyte may result in a slightly thicker CEI since the oxygen peak intensity is slightly lower. As the lattice oxygen is clearly visible for all samples, the CEI thickness should be thinner than the probing depth of ∼10 nm. NMC811 cycled with EC electrolyte shows much higher peak intensities associated with organic oxygen species, phosphorus, and lithium compared to any of the other electrodes. Furthermore, the Ni 3p region in Figure 1d shows a high binding energy shoulder at ∼70 eV for NMC811 with EC electrolyte that is not apparent for the other solvents. This indicates a chemical change in the Ni close to the NMC surface and has previously been observed to coincide with ReSL formation during long-term cycling of NMC811,^52^ potentially corresponding to formation of a Ni–F environment.^53^ These observations indicate that the EC electrolyte is more reactive toward the NMC811 electrode than the other electrolytes, which is in agreement with the electrochemical data shown in Figure 1d,e.

The C 1s spectra in Figure 6b are rather similar for all electrolytes, although the intensity from the carbon black is lower for NMC811 cycled with EC electrolyte. The combination of showing more degradation products, similar CEI thickness on NMC811, and a lower intensity for the carbon black peak suggests that the EC is more prone to react and cover the carbon black.

### Soluble Electrolyte Degradation: Solution-State NMR

**Figure 7:**
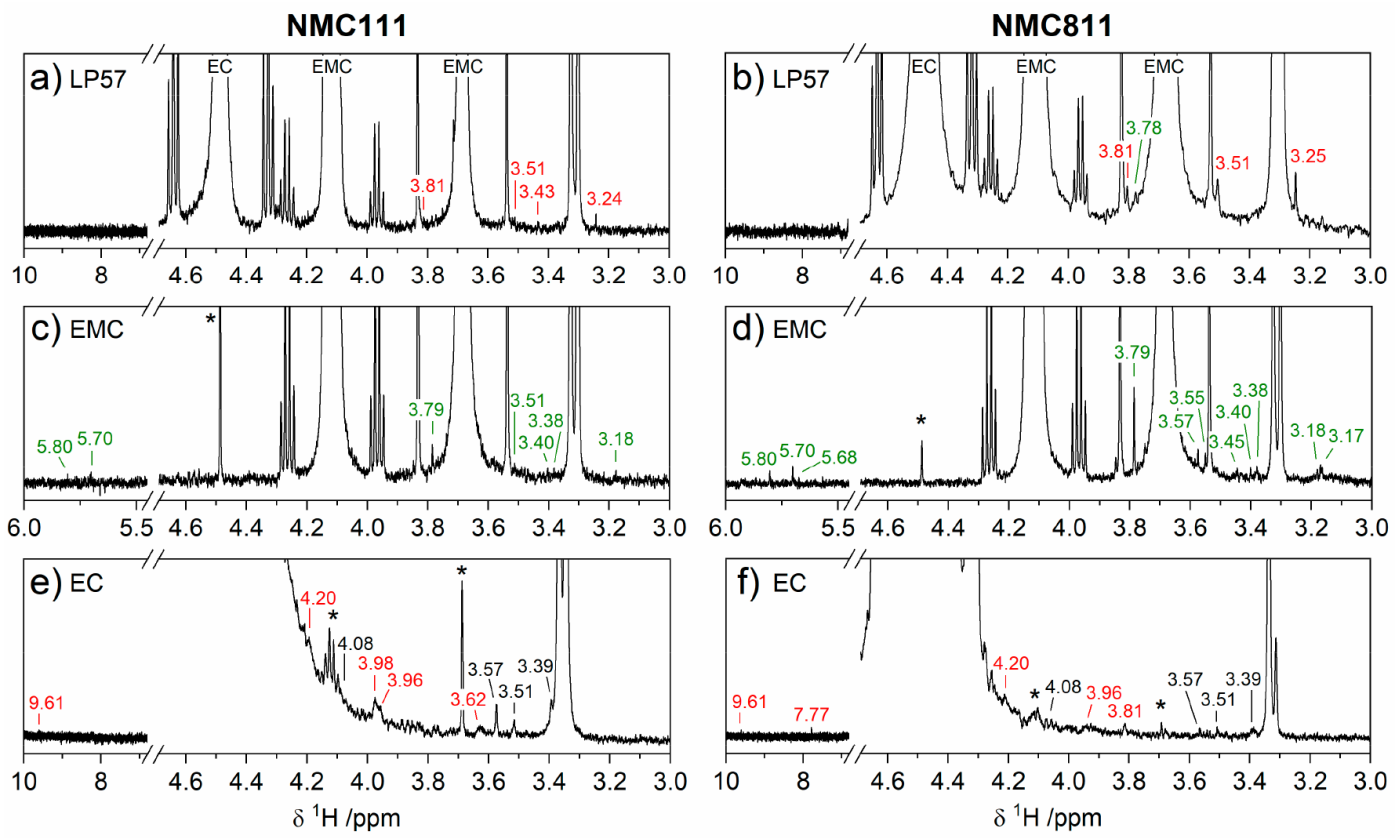
^1^H NMR spectra of the electrolyte extracted from NMC/LTO cells after the first charge–discharge cycle between 1.45 and 3.05 V at C/20 with a 60 h potentiostatic hold at 3.05 V for (left; a,c,e) NMC111 and (right; b,d,f) NMC811 with electrolytes (a,b) LP57, (c,d) 1.5 M LiPF_6_ in ethyl methyl carbonate (EMC), and (e,f) 1.5 M LiPF_6_ in ethylene carbonate (EC). Signals of EC and EMC are annotated in parts a and b. Signals of a trace EC impurity in 1.5 M LiPF_6_ in EMC electrolyte, and a trace EMC impurity in 1.5 M LiPF_6_ in EC electrolyte are marked in parts c–f with an asterisk. The chemical shift labels in black are also present in the pristine electrolyte, while green and red correspond to signals that arise from EMC and EC degradation, respectively.

**Figure 8:**
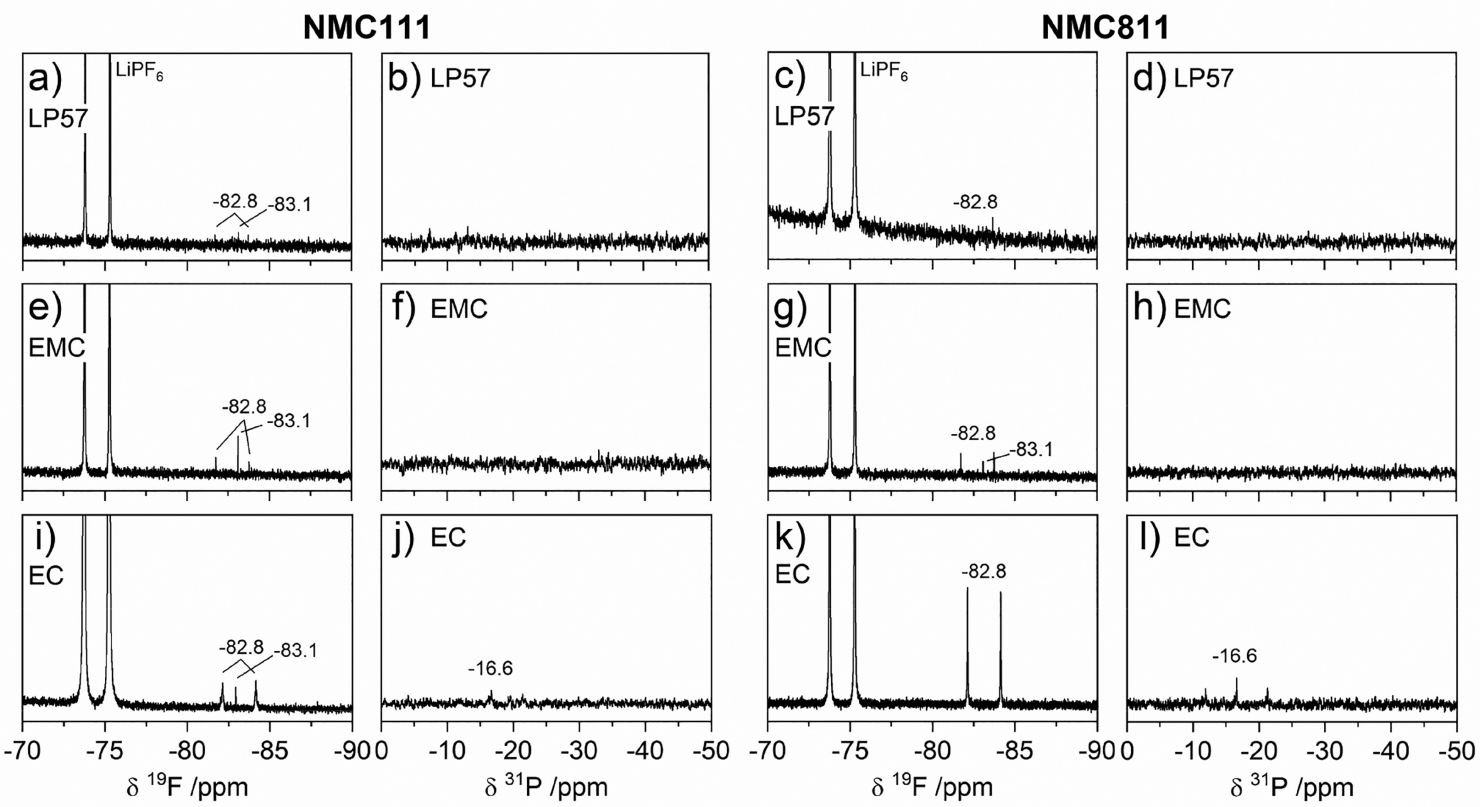
^19^F and ^31^P NMR spectra of the electrolyte extracted from NMC/LTO cells after the first charge–discharge cycle between 1.45 and 3.05 V at C/20 with a 60 h potentiostatic hold at 3.05 V for (left; a,b;e,f;i,j) NMC111 and (right; c,d;g,h;k,l) NMC811 with electrolytes (a–d) LP57, (e–h) 1.5 M LiPF_6_ in ethyl methyl carbonate (EMC), and (i–l) 1.5 M LiPF_6_ in ethylene carbonate (EC). Signals of PF_6_^–^ are annotated in parts a and c.

Pristine electrolyte and the electrolyte extracted from NMC/LTO cells after the 60 h VH protocol were characterized using ^1^H, ^19^F, and ^31^P NMR spectroscopy. Assignments of the observed NMR signals, listed in Table 1, are made based on results reported in the literature.^19,48,54−59^ The ^1^H NMR spectra for deuterated DMSO (the solvent used to extract the electrolyte and degradation products) and the pristine electrolytes are shown in Figures S11 and S12, respectively. Figures 7 and 8 show the ^1^H, ^19^F, and ^31^P NMR spectra of the cycled LP57 (top), EMC electrolyte (middle), and EC electrolyte (bottom) with NMC111 (left) and 811 (right). In Figure 7, signals present in the cycled electrolyte, but absent in the pristine, are labeled in green or red depending on whether they arise from EMC or EC degradation, respectively. The color of the labels for LP57 are based on whether the chemical shift of the particular signal matches with that observed for EMC or EC electrolyte.

**Table 1:** Summary of Observed Chemical Shifts in Pristine and Cycled Electrolytes and the Corresponding Assignments (refs (19, 48, 54−59))^a^

nuclide	chemical shift (ppm)	assignment
^1^H	4.48 (s)	ethylene carbonate (EC)
	4.12 (q, ^3^*J*_H–H_ = 7.1 Hz)	ethyl methyl carbonate (EMC)
	3.69 (s)	EMC
	1.21 (t, ^3^*J*_H–H_ = 7.1 Hz)	EMC
	9.61 (weak)	aldehyde RCHO (e.g., formaldehyde, acetaldehyde, glyoxal)
	2.13 (weak)	acetaldehyde
	7.77 (s)	vinylene carbonate (VC)
	5.80 (s)	acetal RCH(OR)_2_ (e.g., methanediol, 1,1-ethanediol, methoxymethanol, and 1-methoxyethanol)
	5.70 (s)	acetal
	5.68 (s)	acetal
	4.20 (m)	poly ethylene oxide (EO) based oligomers ROCOOCH_2_CH_2_OR′
	3.78–3.81 (s or m)	poly-EO based oligomers
	3.38–3.62 (several m)	poly-EO based oligomers
	3.24 (s)	poly-EO based oligomers
	3.38–3.39 (m)	ethylene glycol
	4.08 (t, ^3^*J*_H–H_ = 4.7 Hz)	lithium ethylene monocarbonate (LEMC)
	3.57 (t, ^3^*J*_H–H_ = 4.7 Hz)	LEMC
	3.98 (s)	oxyfluorophosphate salts
	3.96 (d, ^3^*J*_P–H_ = 10.1 Hz)	OPF_2_(OCH_3_)
	3.34 (s)	water
	3.32 (s)	DMSO impurity
	3.18 (d, ^3^*J*_H–H_ = 5.5 Hz)	methanol
		
^19^F	–74.5 (d, ^1^*J*_P–F_ = 711 Hz)	LiPF_6_
	–82.8 (d, ^1^*J*_P–F_ = 949 Hz)	PO_2_F_2_^–^
	–83.1 (s)	oxyfluorophosphate salts
		
^31^P	–145.0 (septet, ^1^*J*_F–P_ = 711 Hz)	LiPF_6_
	–16.6 (t, ^1^*J*_F–P_ = 949 Hz)	PO_2_F_2_^–^

a The *J*-coupling multiplicity is indicated in parentheses; weak or minor peaks, where the *J*-coupling is less clear, are also indicated.

Starting with the ^1^H NMR of EMC electrolyte (Figure 7c,d), the intense signals at 4.12 ppm (q) and 3.69 ppm (s) are from EMC, while those at 3.34 ppm (s, water) and 3.32 ppm (s), which are present in all pristine and cycled electrolytes, appear to be introduced from impurities in DMSO (see Figure S11). The degradation products identified in EMC electrolyte for NMC111 and 811 are methanol (3.18 ppm, d),^54^ polyethylene oxide (EO) based oligomers likely containing carbonate groups (ROCOOCH_2_CH_2_OR′; multiple peaks in the region 3.38–3.62 ppm; 3.79 ppm, s),^55^ and simple acetal species (RC***H***(OR_2_); possibilities include methanediol, 1,1-ethanediol, methoxymethanol, and 1-methoxyethanol; 5.80 ppm, s; 5.70 ppm, s; 5.68 ppm, s).^19^ A doublet at −82.8 ppm in the ^19^F NMR spectra with NMC111 and 811 (Figure 8e,g) indicates the formation of PO_2_F_2_^–^,^48,56,57^ but the expected triplet at −16.6 ppm in the ^31^P NMR spectra^57^ (Figure 8f,h) is not observed, presumably because the quantity present is below the detection limit. Similar electrolyte degradation products for NMC111 and 811 suggest that the reaction pathways are largely independent of Ni content. However, NMC111 has fewer signals from poly-EO based oligomers and a lower PO_2_F_2_^–^/PF_6_^–^ peak area fraction (Table S5), indicating less EMC solvent decomposition and less LiPF_6_ salt breakdown with the lower Ni cathode.

The ^1^H NMR spectra of EC electrolyte (Figures 7e,f) have an intense signal at 4.48 ppm (s) from EC and a trace EMC impurity (marked with asterisks and also present in the pristine electrolyte, Figure S12). Signals at 4.08, 3.57, 3.51, and 3.39 ppm (labeled in black) are also present in the pristine electrolyte and are attributed to hydrolysis products of EC, specifically LEMC^19^ and poly-EO based oligomers^55^ and/or ethylene glycol.^60^ The degradation signals with NMC111 and 811 are assigned to poly-EO based oligomers likely containing carbonate groups (3.62 ppm, m; 3.81 ppm, m; 4.20 ppm, m),^55^ aldehyde species (RC***H***O; possibilities include formaldehyde, acetaldehyde, and glyoxal; 9.61 and 2.13 ppm, weak),^19,58^ and oxyfluorophosphate salts (3.96 ppm, d; 3.98 ppm, s; e.g., OPF_*x*_(OR)_*y*_).^57,59^ A singlet at 7.77 ppm is observed with NMC811 but absent with NMC111, which has been assigned to vinylene carbonate (VC).^61^ Signals for PO_2_F_2_^–^ are evident in the ^19^F and ^31^P NMR spectra in Figure 8i–l.^48,56,57^ The detection of signal from PO_2_F_2_^–^ in the ^31^P spectra of the EC electrolyte but not the EMC electrolyte, suggests more LiPF_6_ salt decomposition in EC electrolyte. As was found for EMC electrolyte, the reaction pathways for EC electrolyte decomposition appear to be independent of Ni content. Signs of less EC solvent decomposition and less LiPF_6_ salt breakdown with the lower Ni cathode are again seen via fewer signals from poly-EO based oligomers and a lower PO_2_F_2_^–^/PF_6_^–^ peak area fraction (Table S5).

The ^1^H NMR spectra of LP57 (Figure 7a,b) show intense signals at 4.12 ppm (q) and 3.69 ppm (s) from EMC and at 4.48 ppm (s) from EC. The signatures of degradation detected are a subset of those found in the cycled EC and EMC electrolytes and can be assigned to poly-EO based oligomers.^55^ Very weak signals for PO_2_F_2_^–^ are present in the ^19^F spectra with both NMC111 and 811 (Figure 8a,c).

To decouple the species formed, or whose formation is initiated, by chemical oxidation (via reaction with reactive lattice oxygen) and direct electrochemical oxidation pathways, the same electrochemical protocol was applied to a LiMn_2_O_4_ (LMO) cathode which, unlike NMC, does not evolve oxygen at high SOC. The ^1^H NMR spectra of the cycled electrolytes extracted from LMO/LTO cells is shown in Figure S13. Aside from signals also present in the pristine electrolyte, there are no signals observed that are in common with those seen in the cycled electrolytes from NMC/LTO cells. This strongly indicates that the degradation species identified for NMC111 and 811 in Figure 7 are initiated by chemical oxidation, or a coupled chemical and electrochemical pathway; these pathways are discussed further in the Discussion section.

### Transition Metal Dissolution: ICP-OES and XPS

**Figure 9:**
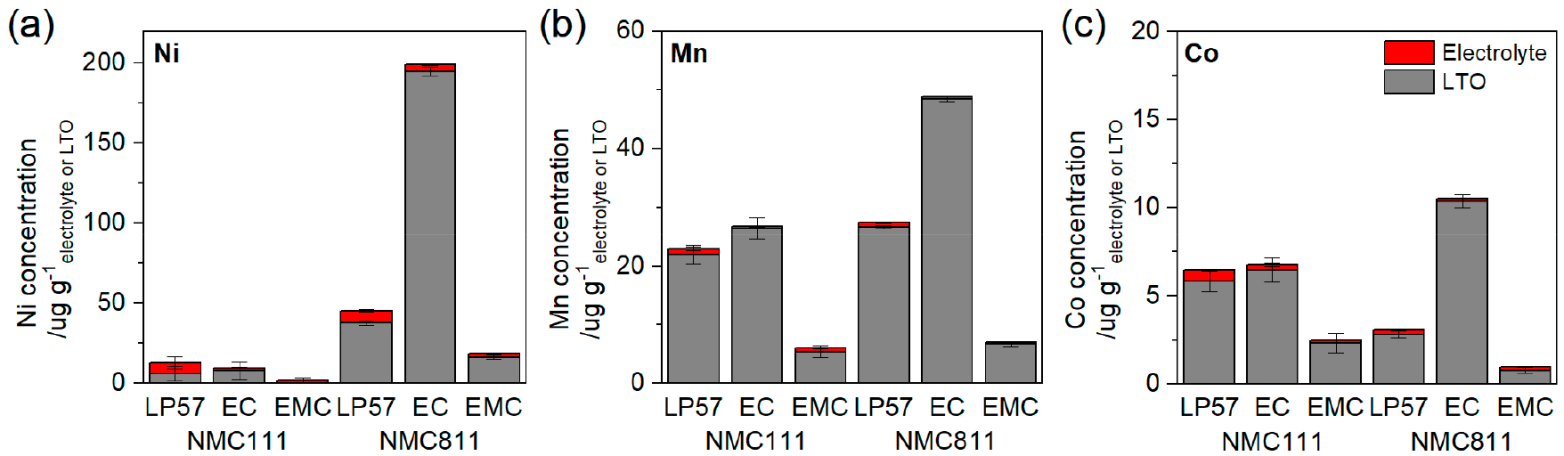
(a) Ni, (b) Mn, and (c) Co concentrations dissolved in the electrolyte and deposited on LTO electrodes extracted from NMC/LTO cells after the first charge–discharge cycle between 1.45 and 3.05 V at C/20 with a 60 h potentiostatic hold at 3.05 V for NMC111 and 811 with electrolytes LP57, 1.5 M LiPF_6_ in ethylene carbonate (EC), and 1.5 M LiPF_6_ in ethyl methyl carbonate (EMC).

To examine the extent of TM dissolution after the 60 h VH protocol, cycled electrolyte and LTO electrodes were extracted from NMC/LTO cells for characterization by ICP-OES. Figure 9 shows the concentration of Ni, Mn, and Co dissolved in the electrolyte and deposited on the LTO anode for NMC111 and 811 and for LP57, EC, and EMC electrolytes; tabulated values are given in Table S6. The values are normalized per mass of electrolyte or LTO extracted from the cell. The concentration of TMs in the electrolyte was significantly lower than the concentration on the LTO anode in most cases. The effect of the NMC composition can be determined by comparison of NMC111 and 811 with LP57, and the higher Ni content of NMC811 must be accounted for. For the pristine NMCs, the TM_811_/TM_111_ fraction for Ni, Mn, and Co in the electrode are (0.8/0.33 =) 2.42, 0.30, and 0.30, respectively. However, with LP57 electrolyte the TM_811_/TM_111_ fraction of dissolved/deposited Ni, Mn, and Co are 3.54, 1.20, and 0.48, respectively. This indicates higher relative quantities of TM dissolution from NMC811 for all three TMs and is 1.5 times higher for Ni, 3.9 times higher for Mn, and 1.6 times higher for Co (Figure S14). TM dissolution is also strongly influenced by the electrolyte. With NMC111, the concentration of Mn and Co are lower in EMC electrolyte than LP57 and EC electrolyte (by ∼0.24 and ∼0.38 times, respectively), while the Ni concentrations are within the error of the measurement. With NMC811, the electrolyte-dependence is more striking. The concentration of Ni, Mn, and Co are 4.39, 1.78, and 3.42 times higher in EC electrolyte than in LP57, while in EMC the concentration of Ni, Mn, and Co are lower, 0.41, 0.25, and 0.32 times that in LP57.

The XPS spectra of the LTO electrodes (paired with NMC811 cathodes) after cycling in different electrolytes (Figure S15) are in agreement with the ICP-OES results. Specifically, the intensities of the Ni 3p, Co 3p, and the Mn 3p are highest for the LTO electrode cycled in EC electrolyte while barely any TMs are detected with EMC electrolyte. In the literature, TM dissolution has been associated with corrosion of NMC by dissolved H^+^ and HF,^62,63^ the solvating power of the solvent,^64,65^ and lattice oxygen release,^66^ as explored further in the Discussion section.

## Discussion

We first revisit the average current measured in the final 20 h of the 4.6 V vs Li/Li^+^ potentiostatic hold. We propose that the electrolyte-dependent current measured with NMC811 (Figure 1e) is primarily due to the electrolyte-dependent lattice oxygen release (Figures 2 and 3a), with the current and the quantity of (measured/inferred) oxygen released increasing in the order EMC electrolyte, LP57, and EC electrolyte. Such electrolyte-dependence is not seen with NMC111 seemingly due to the lower lattice oxygen release at 4.6 V vs Li/Li^+^ (Figure 2 and Figure S5). This suggests that EC and EMC have similar interfacial reactivity when NMC is below the onset potential of lattice oxygen release, but above this potential the measured current is higher for EC-containing electrolytes as EC promotes, or is less effective at preventing, oxygen loss relative to EMC. Note that while direct electrochemical oxidation of the electrolyte may contribute to the measured current, the current for NMC111 in each electrolyte provides an upper limit for its contribution.

The oxygen released during the voltage hold can either be detected directly as oxygen gas or via products of further chemical reactions with the electrolyte. The high fraction of CO + CO_2_ evolved, relative to oxygen, particularly for NMC811, indicates that most of the oxygen must react chemically with the electrolyte. Gasteiger and co-workers have proposed that the chemical oxidation reaction of EC with reactive lattice oxygen yields CO_2_, CO, and H_2_O (EC + 2O_2__(lattice)_ → 2CO_2_ + CO + 2H_2_O).^6^ Based on calculations, they also proposed a pathway including the formation of VC (EC + O_2 (lattice)_ → VC + H_2_O_2_), where VC can further react forming CO_2_ and CO.^11^ We observe a signal at 7.77 ppm, consistent with VC, in the ^1^H NMR spectra of cycled EC electrolyte with NMC811, suggesting that an EC to VC reaction is occurring at the charged NMC interface. Shao-Horn and co-workers have also recently reported the dehydrogenation of EC forming VC at the charged NMC811 interface using in situ Fourier-transform infrared spectroscopy.^67^

**Scheme 2:**
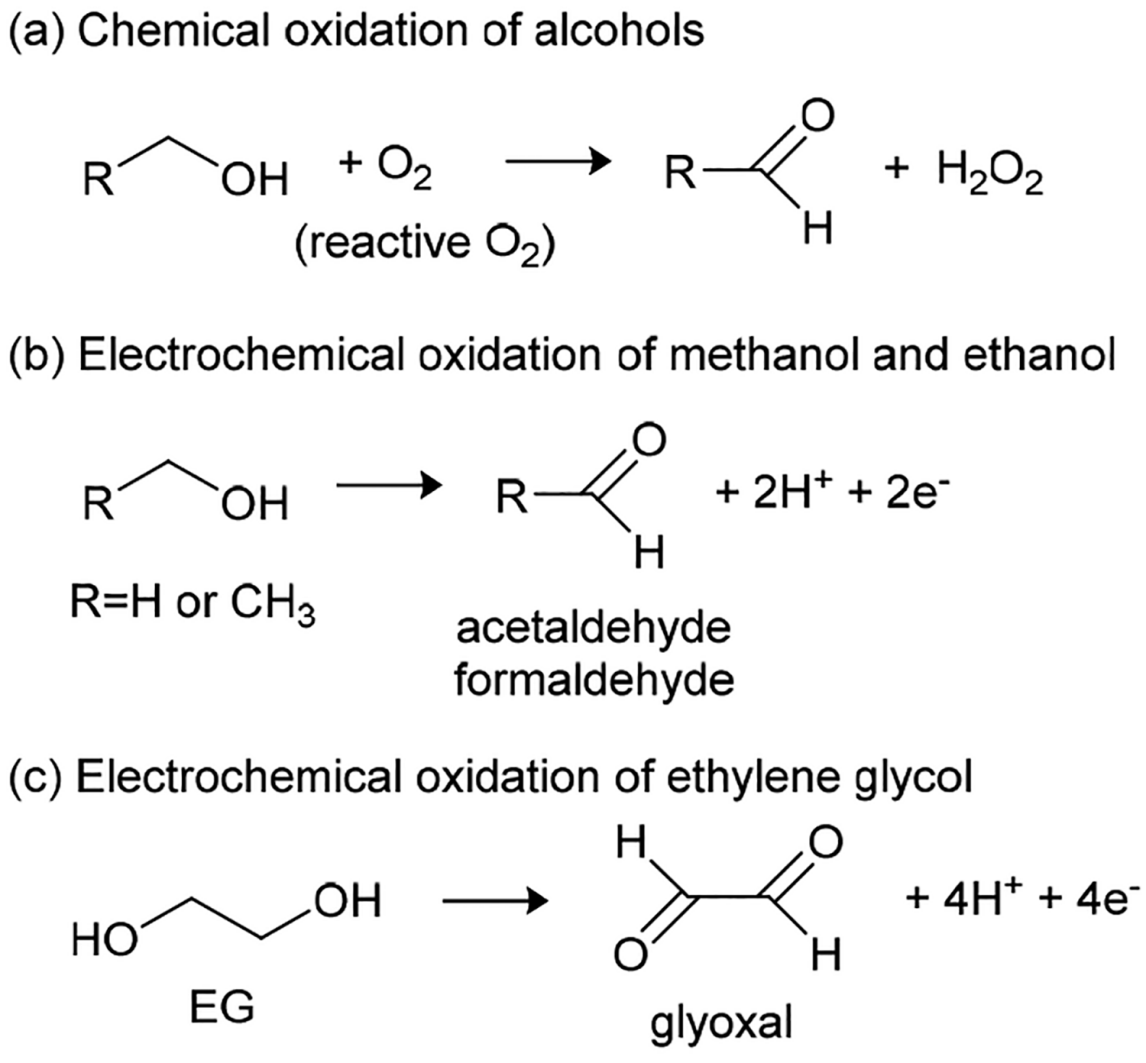
Chemical and Electrochemical Oxidation of Alcohols to Aldehydes

The enhanced gassing measured with NMC811 and EMC electrolyte (compared with NMC111) proves that reactive lattice oxygen will also react with EMC producing CO_2_ and CO (Figures 2 and 3a), albeit with a reduced CO_2_/CO ratio compared to EC electrolyte (Figure 3c). While multiple reaction pathways are possible with a highly reactive species such as singlet oxygen, we propose a possible reaction mechanism in Scheme 1. Most importantly, the overall stoichiometry of the reaction is EMC + O_2__(lattice)_ → EtOH + CO_2_ + CO + H_2_O. Ethanol will be unstable both chemically, in the presence of reactive lattice oxygen, and electrochemically, at the potential of the NMC, being oxidized to acetaldehyde and either peroxide (chemical, Scheme 2a) or protons (electrochemical, Scheme 2b). Further reactions involving these products are discussed below.

From the chemical oxidation reaction mechanisms proposed for EC^6^ and EMC (Scheme 1), the expected O_2_/CO_2_ mole ratio is 1:1 for EC and EMC, while the O_2_/CO ratio is 2:1 for EC and 1:1 for EMC. For equal amounts of O_2_ released from the lattice, we would therefore expect that the ratio of CO_2_ and CO evolved for EC relative to EMC to be 1 and 1/2, respectively. Instead, we measure EC/EMC relative gas evolution fractions of 1.9 for CO_2_ and 0.8 for CO (Figure 3b). Therefore, with NMC811 at 4.6 V vs Li/Li^+^, the amount of CO_2_ and CO released, which is due to the chemical oxidation of the electrolyte solvent, is 1.6–1.9 times higher with EC compared to EMC. This is consistent with the observed direct evolution of O_2_ that is 1.6 times more with EC (Figure 3b); although it should be noted that the measured O_2_ arises from release of ground state O_2_ from the NMC lattice and/or the deactivation of ROS to ground state triplet O_2_—it does not directly measure of the amount of ROS released from NMC. Despite LP57 having a higher volume fraction of EMC, the quantity of evolved CO_2_ and CO closely resembles that with EC electrolyte. This is ascribed to a number of different factors: EC coordinates with PF_6_^–^ more strongly and thus reaches the NMC surface more easily during charging,^4,48,68^ EC has a poorer chemical stability toward reactive lattice oxygen compared to linear carbonates,^11^ and finally EC is likely to more strongly coordinate to TM ions (see below), promoting dissolution and concomitant O_2_ loss.

Oxygen loss is required for the structural reconstruction from layered LiMO_2_ to rock-salt (MO) reported to take place at the surface of NMCs.^5^ This process may occur or involve spinel-like structures (M_3_O_4_) which, depending on the stoichiometry, may also involve oxygen loss. The resulting surface layer is believed to be the primary driver for NMC impedance rise due to the poorer lithium transport across this interface.^5,69^ Therefore, the higher NMC811 impedance measured with LP57 and EC electrolyte compared to EMC electrolyte (Figure 4e,f) corroborates the OEMS finding of more lattice oxygen release in EC-containing electrolytes. This is also supported by the HRTEM (Figure 5), which revealed a thick rock-salt SRL on the NMC811 particles with EC-containing electrolytes, while a thinner (3–5 nm) SRL having both a rock-salt layer and a cation mixing layer forms with EMC electrolyte. Therefore, characterization by OEMS, EIS, and HRTEM all support the conclusion of a higher amount of lattice oxygen release from NMC in EC-containing electrolytes. This combined experimental approach highlights that the electrolyte solvent has a profound influence on the Ni-rich NMC interfacial degradation from very early in the cycle life.

**Scheme 3:**
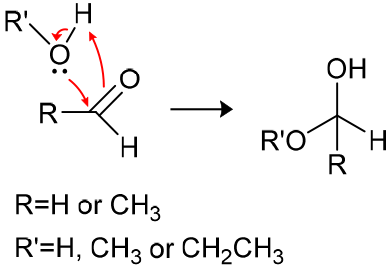
Nucleophilic Attack of Aldehydes by Water or Alcohols to Form Acetals

In addition to CO_2_ and CO evolution, chemical oxidation of EC and EMC also produces water (ref (6) and Scheme 1, respectively), which initiates a number of degradation processes. First, water can react with the carbonate solvents, with evidence for the hydrolysis products of EC and EMC, such as LEMC, ethylene glycol, poly-EO based oligomers, and methanol (see Scheme S1), detected in the ^1^H NMR spectra of cycled electrolytes (Figure 7). EC has been reported to be more susceptible toward hydrolysis than EMC,^70^ compounding the instability of EC-containing electrolytes in the presence of an oxygen-releasing cathode. The alcohols formed by solvent hydrolysis (i.e., methanol, ethanol, and ethylene glycol; Scheme S1) and/or solvent chemical oxidation (i.e., ethanol; Scheme 1) can be chemically or electrochemically oxidized, as shown in Scheme 2, forming aldehydes (consistent with the ^1^H NMR assignments above) and either peroxide or protons. The oxidation potential of these alcohols, and the peroxide formed, have been reported by Gasteiger and co-workers to be ∼3.5–4.0 V and ∼3.85 V vs Li/Li^+^, respectively,^11,71^ and are therefore unstable at the potentials for lattice oxygen release, i.e., above 4.4 V vs Li/Li^+^ for NMC811. The aldehydes formed in Scheme 2 may further react via nucleophilic attack by water or alcohols to form acetals, as shown in Scheme 3, which were observed by ^1^H NMR in the cycled EMC electrolyte (Figure 7d).

**Scheme 4:**
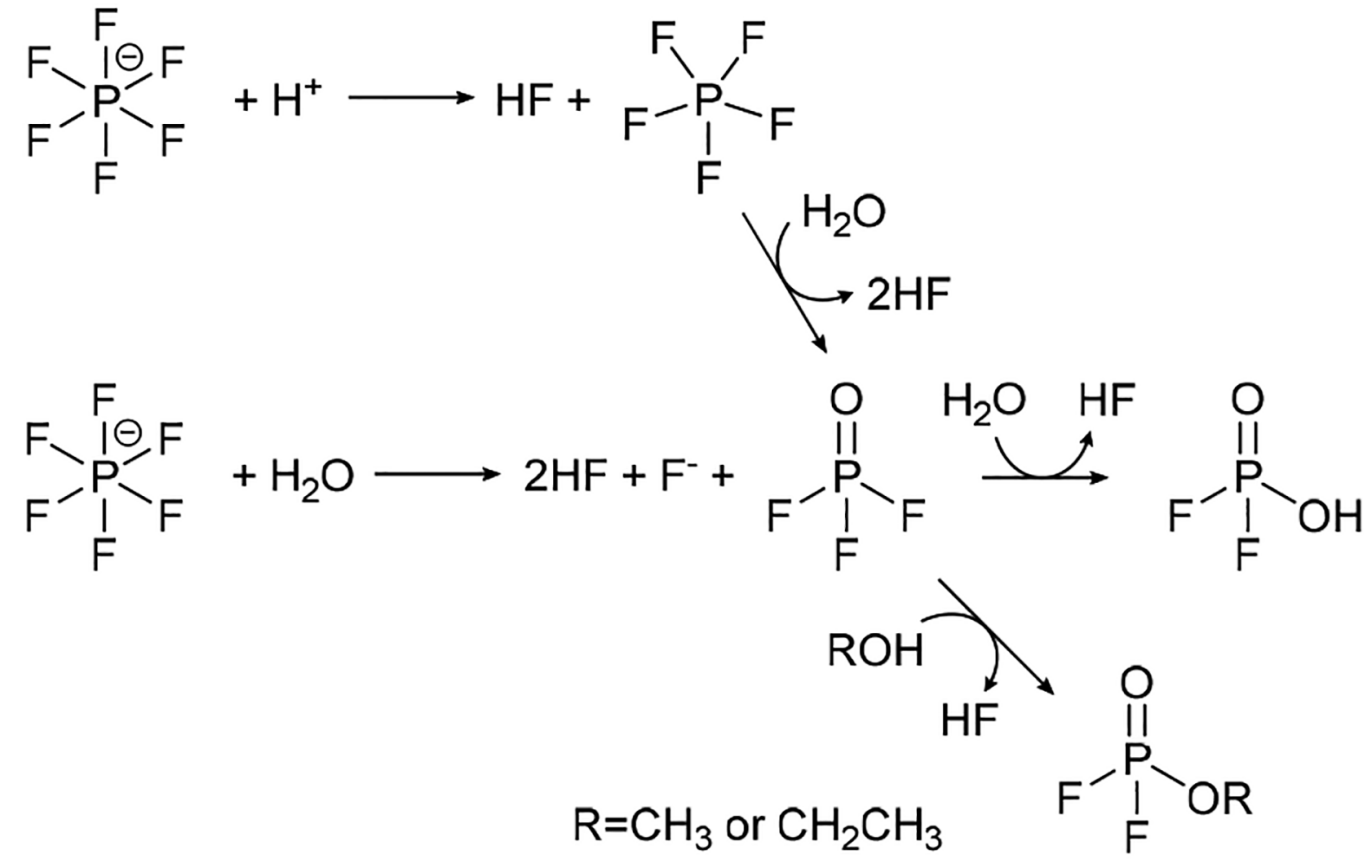
LiPF_6_ Salt Decomposition^57,59^

Second, the water and protons concomitant with lattice oxygen release both enhance the decomposition of the LiPF_6_ salt, as shown in Scheme 4.^57,59^ Evidence for PO_2_F_2_^–^ is observed in the ^19^F and ^31^P NMR spectra in Figure 8 and for Li_*x*_PO_*y*_F_*z*_ in the XPS spectra in Figure 6. Interestingly, in the NMR spectra, we observe higher levels of *soluble* salt decomposition products with NMC811 vs 111 (Figure 8 and Table S5) but lower levels of *insoluble* products close to the CEI surface with NMC811 vs 111 in the P 2p XPS spectra (Figure 6c and Figure S10), which is in agreement with recently reported findings by Yu et al.^48^ The deposited salt decomposition products on NMC811 may be dissolved at high SOC, possibly promoted by the higher concentrations of acidic protons and greater oxygen loss (resulting in an unstable surface), thus exposing the Ni-rich NMC surface to further electrolyte solvent and salt breakdown. In addition, we observe clear electrolyte-dependent salt decomposition behavior with Ni-rich NMC811. Higher levels of salt decomposition are noted in both the NMR spectra (Figure 8 and Table S5) and XPS spectra (Figure 6) for EC vs EMC electrolyte, which can be rationalized by the enhanced lattice oxygen release and hence water production with EC electrolyte. It is also worth noting that while EC promotes LiPF_6_ degradation at Ni-rich cathode surfaces, EC-free electrolytes lead to more LiPF_6_ degradation at graphite anodes,^72^ due to formation of a less protective SEI; i.e., the effect of the solvent on the salt breakdown is electrode/cell chemistry dependent.

Third, water, protons, and/or other protic species formed at the NMC at high SOCs migrate to the negative electrode where they can react. In a LIB with a graphite anode, acidic species have been reported to decompose or react with components of the SEI^73^ and as such they likely contribute significantly to capacity loss. In addition, water and protic species can be electrochemically reduced to H_2_, the H_2_ being detected in the OEMS measurements (Figure 2). The OH^–^ formed as a side-product of water reduction (H_2_O + e^–^ → 1/2H_2_ + OH^–^) could initiate OH^–^ driven hydrolysis of EC and EMC (Scheme S1), which proceeds much faster than water driven hydrolysis at 25 °C since OH^–^ is a stronger nucleophile.^47^ These reactions, which produce CO_2_ but not CO, may account for the higher measured CO_2_/CO fraction (6.9 for EC and 3.0 for EMC, Figure 3c) than expected: 2 for EC^6^ and 1 for EMC (Scheme 1). Note that electrochemical oxidation of EC and EMC may also contribute to the observed H_2_ evolution (through reduction of protic species formed,^68,74^ see Scheme S2) and to the higher fraction of CO_2_ since CO is less stable at these highly oxidative potentials.^68^ We also note that CO may itself by oxidized directly by ROS to form CO_2_ (CO + O _(lattice)_ → CO_2_). Since water reduction has been shown to take place at LTO potentials (1.55 V vs Li/Li^+^),^75^ any reaction mechanisms initiated by water reduction are relevant for battery chemistries with LTO, graphite, silicon, and Li metal anodes.

The trends in lattice oxygen release, and in particular the follow-up reactions with the electrolyte, have significant implications for TM dissolution with Ni-rich NMC cathodes, which is a key driver of capacity loss in LIBs with graphite anodes.^18^ Starting with NMC111, the similar TM dissolution/deposition for LP57 and EC electrolyte (Figure 9) suggests that with little or no lattice oxygen release, the properties of EC dominate the LP57 behavior. The lower TM dissolution/deposition for EMC electrolyte could be related to the relative solvating ability of EC versus EMC and thus the increased stabilization of the TMs by the EC in the electrolyte. It has been established that Li^+^ are preferentially solvated by EC over EMC; the solvating power of EC is 1.41 times that of EMC.^64^ Similarly, Wang et al.^65^ report that Mn^2+^ in the electrolyte prefers EC over EMC (the interaction energy calculated by DFT is more negative), with a similar trend likely extending to Ni^2+^ and Co^2+^, which may rationalize the higher TM dissolution in EC-containing electrolytes.

The higher relative amounts of TM dissolution/deposition with NMC811 for all three electrolytes (compared to NMC111 in the respective electrolyte and taking into account the relative TM fractions, Figure 9 and Figure S14) are likely associated with the lattice oxygen release-induced H_2_O and H^+^ formation, as discussed above. These species decompose PF_6_^–^ forming HF (Scheme 4), which leads to etching of the NMC cathode and TM dissolution.^62^ Similar conclusions have been reached by Gasteiger and co-workers.^20,76^ Oxygen release also leads to under coordinated TMs at the surface of NMC, which will be easier to dissolve.^52,77^ Furthermore, the strong electrolyte dependence, and specifically the significantly higher TM dissolution/deposition for EC electrolyte, is consistent with the enhanced lattice oxygen release (and hence H_2_O and H^+^ formation) observed for EC electrolyte compared to EMC electrolyte. The heightened susceptibility for EC vs EMC toward hydrolysis driven degradation^70^ will likely also play a role in the overall TM dissolution/deposition due to acidification of the electrolyte (Scheme S1 and Scheme 2). Further, the relative stabilizing influence of the carbonate solvent and/or electrolyte degradation products in solvating TMs in solution may also contribute to this effect. For instance, more TM dissolution/deposition for EC electrolyte is consistent with (i) EC having a higher solvating ability compared to EMC^64^ and (ii) recent reports of degraded LiPF_6_ species, which are present in greater quantities with NMC811 in EC electrolyte than in EMC electrolyte (Figure 8 and Table S5), preferentially coordinating with TMs in the electrolyte.^72^ These effects may rationalize the lower TM dissolution/deposition observed in LP57 compared to EC electrolyte with NMC811, despite these two electrolytes exhibiting strong similarities in the NMC811 gassing behavior (i.e., quantities of CO_2_ and CO evolved, Figure 3) and impedance (Figure 4), as noted above.

## Conclusions

In this work, investigation of low- and high-Ni content NMC with single solvent LiPF_6_-based electrolytes (EC-only and EMC-only) has unlocked new understanding of the increased interfacial reactivity for charged Ni-rich NMC cathodes and the pivotal role played by the electrolyte solvent. The benefit of this approach is that the reactivity of each carbonate solvent is determined separately, providing an improved understanding of standard LIB electrolytes, which are a mixture of two or more carbonate solvents.

A key finding herein is that the degree of lattice oxygen loss from NMC, which was found to be dependent on both the Ni-content and the electrolyte solvent, is intrinsically linked to the degradation of the cathode surface and subsurfaces and electrolyte solvent and salt. In particular, with a Ni-rich NMC cathode, electrolytes containing EC were shown to lead to more oxygen loss, more extensive cathode surface layer reconstruction, higher cathode interfacial impedance, more electrolyte solvent and salt decomposition, and higher amounts of transition metal dissolution, compared to that with a single solvent EMC-based electrolyte.

The present work provides critical mechanistic insights that shed new light on recent reports of superior long-term cycling performance of EC-free electrolytes, containing interphase-forming additives, with high-Ni content cathodes^2^ and with low-Ni NMC cathodes operating at high potential.^1,22^ It is worth noting that EC plays a critical role in the performance and safety of current-generation LIBs, related to its role in SEI formation/repair, has a higher ionic conductivity of LiPF_6_ in EC vs linear carbonates, and has the ability to inhibit severe gassing in the case of Li plating on the graphite. It is hoped that the compatibility issues between EC-containing electrolytes and Ni-rich cathodes identified in this work will direct future research to critically assess the role of EC in future LIB electrolytes and inspire studies of the long-term cycling performance, interfacial reactivity, and safety of new electrolytes with less, or even without, EC. Finally, this work has provided fundamental understanding to facilitate the rational design of novel electrolyte chemistries and material coatings that stabilize the cathode–electrolyte interface, which is an important step toward enabling LIBs with high capacity Ni-rich cathodes.

## Supporting Information Available

Experimental setup; electrochemical data; BET surface area data; Karl Fischer titration data; additional OEMS data; additional XPS spectra; additional ^1^H NMR spectra and assignments; tabulated ICP data; and reaction schemes for hydrolysis and electrochemical oxidation of carbonate solvents (PDF)
