# Supplementary material for: Electrolyte Reactivity at the Charged Ni-Rich Cathode Interface and Degradation in Li-Ion Batteries
Source: ACS Appl Mater Interfaces. 2022 Mar 8;14(11):13206–22. doi: 10.1021/acsami.1c22812 (PMC9098117; doi:10.1021/acsami.1c22812)
Supplement: Supplementary file 1 — am1c22812_si_001.pdf [file am1c22812_si_001.pdf]

Electrolyte reactivity at the charged Ni-rich cathode interface and degradation in Li-ion batteries

Wesley M. Dose[1,2,3], Israel Temprano[2], Jennifer P. Allen[2,3], Erik Björklund[3,4], Christopher A. O'Keefe[2,3], Weiqun Li[3,5], B. Layla Mehdi[3,5], Robert S. Weatherup[3,4], Michael F. L. De Volder[1,3,*], Clare P. Grey[2,3,*]

[1]Department of Engineering, University of Cambridge, 17 Charles Babbage Road, CB3 0FS, Cambridge, UK.

[2]Department of Chemistry, University of Cambridge, Lensfield Road, Cambridge, CB2 1EW, Cambridge, UK.

[3]The Faraday Institution, Quad One, Harwell Science and Innovation Campus, Didcot OX11 0RA, UK.

[4]Department of Materials, University of Oxford, Parks Road, Oxford OX1 3PH, UK.

[5]Department of Mechanical, Materials and Aerospace Engineering, University of Liverpool, Liverpool L69 3GH, UK.

*Correspondence: Clare P. Grey (cpg27@cam.ac.uk), Michael F. L. De Volder (mfld2@cam.ac.uk).

Supporting information

Table S1. Surface area of NMC powders.

NMC	BET surface area /m^2 g^{-1}
NMC111	0.435
NMC811	0.275

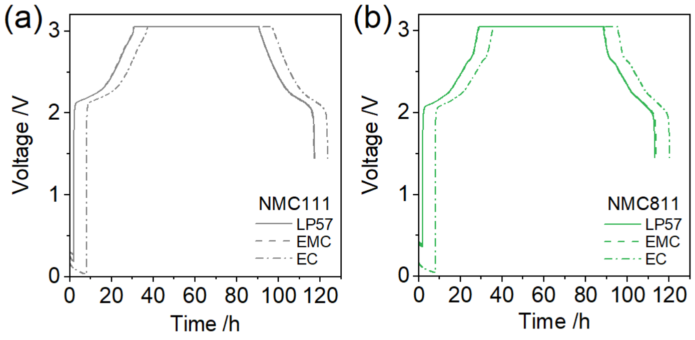

Figure S1. Representative voltage profiles for NMC/LTO coin cells during the first charge-discharge cycle between 1.45–3.05 V at C/20 with a 60 h potentiostatic hold at 3.05 V for (a) NMC111 and (b) NMC811 with electrolytes LP57, 1.5 M $LiPF_6$ in ethyl methyl carbonate (EMC), and 1.5 M $LiPF_6$ in ethylene carbonate (EC).

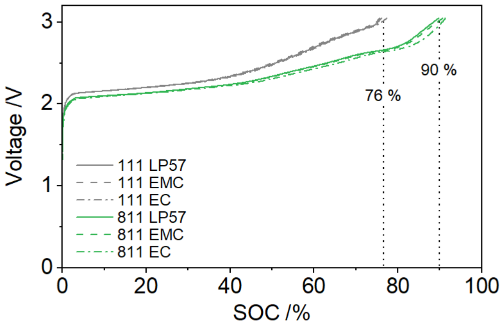

Figure S2. Representative voltage profiles for NMC/LTO coin cells during the first charge to 3.05 V at C/20 for NMC111 and NMC811 with electrolytes LP57, 1.5 M $LiPF_6$ in ethyl methyl carbonate (EMC), and 1.5 M $LiPF_6$ in ethylene carbonate (EC) plotted versus the NMC state-of-charge (SOC). The NMC SOC is calculated from the electrochemistry and assumes a theoretical capacity of 277.9 mAh g^{-1} for NMC111 and 275.5 mAh g^{-1} for NMC811.

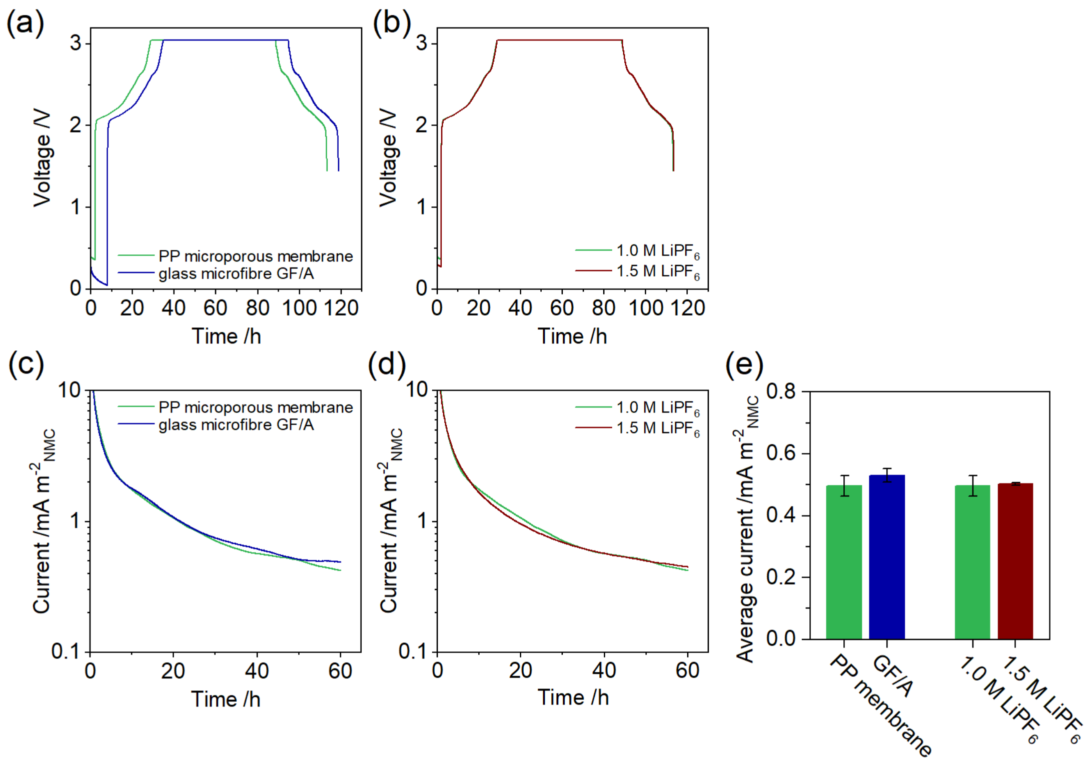

Figure S3. (a-b) Representative voltage profiles for NMC/LTO coin cells during the first charge-discharge cycle between 1.45–3.05 V at C/20 with a 60 h potentiostatic hold at 3.05 V with different (a) separators (polypropylene (PP) microporous membrane and glass microfiber grade GF/A) and (b) $LiPF_6$ salt concentrations (1.0 and 1.5 M) in ethylene carbonate/ethyl methyl carbonate (EC/EMC) 3/7 by volume. (c-d) Corresponding oxidation current during the potentiostatic hold, and (e) the average current in the final 20 h of the potentiostatic hold. Error bars in (e) represent the spread of 2 duplicate cells.

Table S2. Water content in electrolytes.

Electrolyte	Water content /ppm-v
LP57	30
1.5 M $LiPF_6$ in EMC	37
1.5 M $LiPF_6$ in EC	37

Supplementary note S1. Cell chemistry for the OEMS experiment.

LTO and NMC electrodes are both known to generate gases during cycling in carbonate electrolytes. For LTO these include H_2, CO, CO_2, and various hydrocarbons,[1,2] while for NMC these are mainly O_2, CO, and CO_2.[3–5] The overlap means that in a NMC/LTO cell the deconvolution of the gases originating at the cathode and anode would be challenging. To circumvent this, a NMC/Li half-cell is used for the OEMS experiments. Li metal electrodes also produce gas due to electrolyte reduction and SEI formation (mainly CO_2 but also CO and various hydrocarbons)[6] both on contact with the electrolyte (before the start of OEMS experiment – not detected) and during Li plating/stripping. However, with a constant potential and low current density, only small and constant gas production is expected, which is either below the detection limits or removed in signal processing. H_2 evolution has also been reported at Li metal electrodes, attributed to reduction of trace water ($H_2O + e^- \rightarrow 1/2H_2 + OH^-$), reduction of trace HF in the presence of Li^+ ($HF + e^- + Li^+ \rightarrow 1/2H_2 + LiF$), or direct reaction between trace water and Li ($Li + H_2O \rightarrow 1/2H_2 + LiOH$).[7] These reactions are expected to be potential-independent and continue at a steady rate until the reactants are consumed.

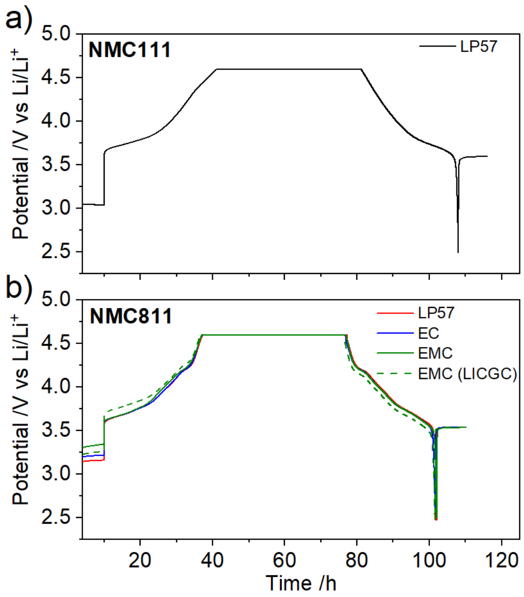

Figure S4. Potential profiles for the NMC/Li OEMS cells during the first charge-discharge cycle between 2.5–4.6 V at C/20 with a 40 h potentiostatic hold at 4.6 V with (a) NMC111 and LP57, and (b) NMC811 and electrolytes LP57, 1.5 M $LiPF_6$ in ethylene carbonate (EC), and 1.5 M $LiPF_6$ in ethyl methyl carbonate (EMC). The potential profile for a NMC811/Li cell with a lithium ion conducting glass-ceramic separator (Ohara, LICGC) with 1.5 M $LiPF_6$ in EMC as the catholyte and LP57 as the anolyte is also shown in (b).

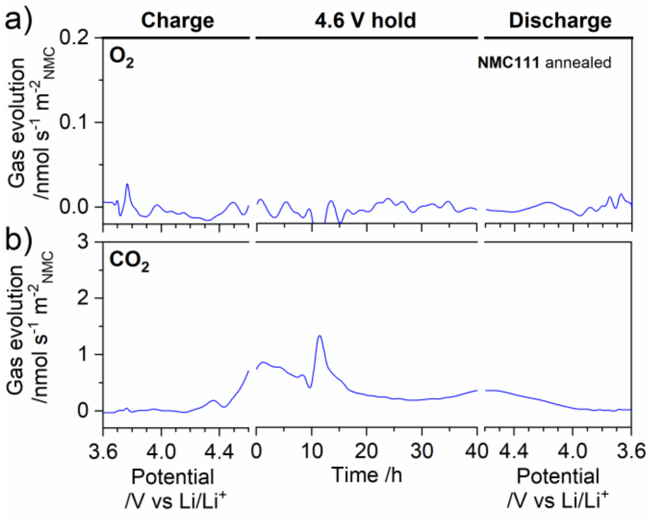

Figure S5. Evolution of (a) O_2 and (b) CO_2 as determined from the OEMS channels m/z = 32 and 44, respectively, and normalized to the NMC surface area, for NMC/Li cells with NMC111 electrodes prepared from freshly annealed powder and LP57 electrolyte during the first charge-discharge cycle between 2.5–4.6 V at C/20 with a 40 h potentiostatic hold at 4.6 V. Data are plotted as a function of potential for the charge and discharge, and time for the potentiostatic hold.

Supplementary note S2. Comparison of the H_2 gas quantity expected from electrochemical reduction of trace water in electrolyte and the H_2 gas quantity measured in the OEMS experiment.

Example calculation for H_2 gas quantity expected from electrochemical reduction of trace water in LP57:

We have,

Volume of electrolyte = 300 μL

LP57 H_2O content = 30.3 ppm-v (see Table S2)

Therefore,

$$\text{Volume of } H_2O \text{ in electrolyte} = 300 \times 30.3\times10^{-6}$$

$$= 9.09\times10^{-3}\ \mu L$$

Mass of H_2O in electrolyte = 9.06×10^{-6} g (at 25 °C)

Moles of H_2O in electrolyte = 9.06×10^{-6} / 18.01528

= 0.503 μmol

From the electrochemical reduction of H_2O to H_2 ($H_2O + e^- \rightarrow 1/2H_2 + OH^-$) we have,

Moles of H_2 evolved = ½ moles of H_2O in electrolyte

= 0.252 μmol

Similarly, for EC and EMC electrolytes:

EC electrolyte: Moles of H_2 evolved = 0.304 μmol

EMC electrolyte: Moles of H_2 evolved = 0.308 μmol

Tabulating the expected quantity of H_2 gas evolution from reduction of trace water in the electrolyte with the quantity of H_2 evolution measured in the OEMS experiment in Figure 2 and 3 yields the following:

Table S3. Measured H_2 evolution in the OEMS experiment compared to that expected from reduction of trace H_2O in the electrolyte.

Electrolyte	Expected H_2 from trace H_2O reduction /μmol	Measured H_2 evolution /μmol	Factor increased
LP57	0.252	NMC111: 18.5	73
		NMC811: 15.4	61
1.5 M $LiPF_6$ in EC	0.304	NMC811: 10.1	33
1.5 M $LiPF_6$ in EMC	0.308	NMC811: 58.1	188

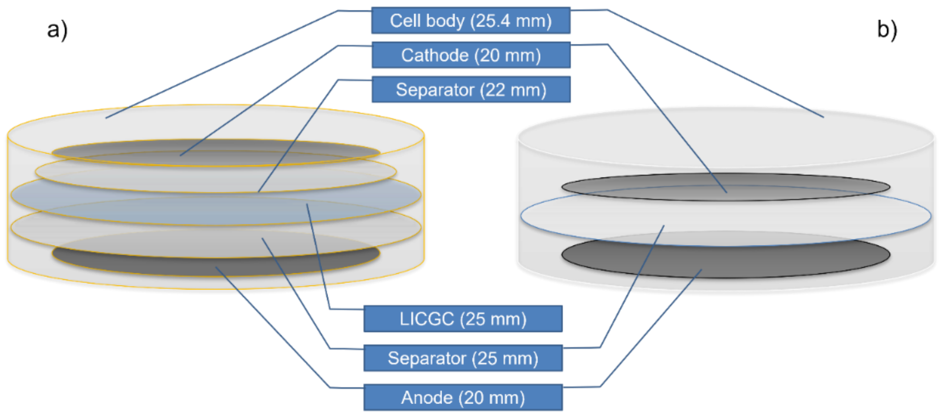

Figure S6. Schematic of the OEMS cell stack (a) with and (b) without a lithium ion conducting glass-ceramic separator (Ohara, LICGC).

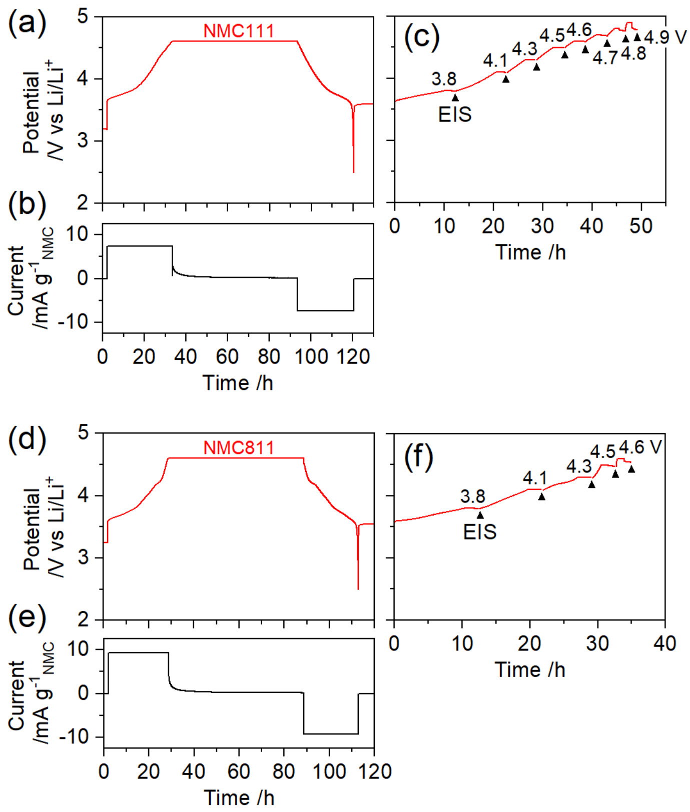

Figure S7. Representative (a, c, d, f) NMC potential and (b, e) current profiles for a three-electrode NMC/LTO cell with a Li metal reference electrode during the (a–b, d–e) first charge-discharge cycle between 2.5–4.6 V at C/20 with a 60 h potentiostatic hold at 4.6 V, and the (e, f) subsequent charge with intermittent potentiostatic holds, OCP periods, and electrochemical impedance spectroscopy (EIS) measurement at the NMC potentials indicated.

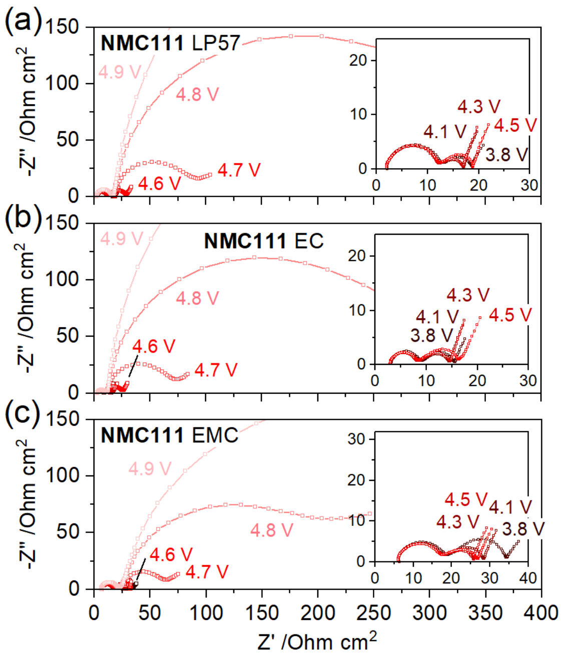

Figure S8. Nyquist impedance plots of the NMC cathode as a function of potential measured in a three-electrode NMC/LTO cell with a Li metal reference electrode after the first charge-discharge cycle between 2.5–4.6 V at C/20 with a 60 h potentiostatic hold at 4.6 V. For NMC111 with electrolytes (a) LP57, (b) 1.5 M $LiPF_6$ in ethylene carbonate (EC), and (c) 1.5 M $LiPF_6$ in ethyl methyl carbonate (EMC).

Table S4. NMC potential and SOC for the Nyquist impedance plots in Figure 4a-d and Figure S8.

NMC	Electrolyte	NMC potential /V	$Li_{1-x}TMO_2$
NMC111	LP57		0.38; 0.60; 0.69; 0.79; 0.84; 0.93; 0.98; 1.0
	1.5 M $LiPF_6$ in EC	3.8; 4.1; 4.3; 4.5; 4.6; 4.7; 4.8; 4.9	0.37; 0.59; 0.69; 0.78; 0.83; 0.90; 0.95; 0.98
	1.5 M $LiPF_6$ in EMC		0.40; 0.62; 0.71; 0.81; 0.87; 0.94; 1.0; 1.0
NMC811	LP57		0.43; 0.66; 0.84; 0.89; 0.90
	1.5 M $LiPF_6$ in EC	3.8; 4.1; 4.3; 4.5; 4.6	0.45; 0.69; 0.86; 0.91; 0.92
	1.5 M $LiPF_6$ in EMC		0.45; 0.69; 0.85; 0.90; 0.92

Supplementary note S3. Determining the electrolyte-oxide interfacial impedance from the EIS data.

Figure S9 shows Nyquist plots of the NMC811 cathode impedance at various potentials vs Li/Li^+. The spectra are reproduced from Figure 4b and were measured in a NMC811/LTO cell with LP57 electrolyte with respect to a lithium metal reference electrode. The high frequency semicircle (hf), mid-frequency semicircle (mf), and Warburg impedance tail at low frequencies (lf) are labelled in Figure S9a.

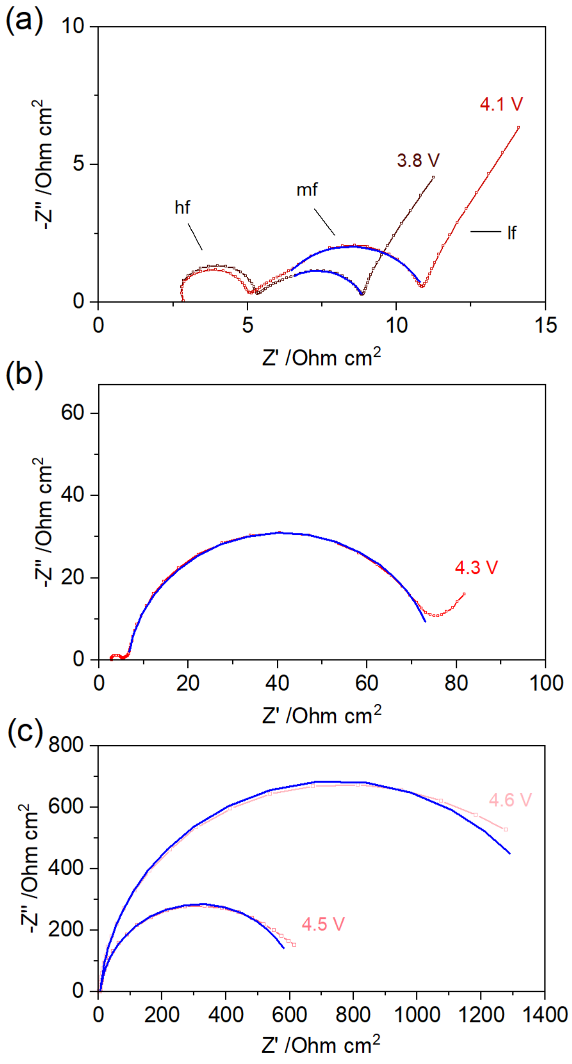

Figure S9. Exemplary Nyquist impedance plots (red) of the NMC cathode as a function of potential ((a) 3.8 and 4.1 V, (b) 4.3 V, and (c) 4.5 and 4.6 V) measured in a three-electrode NMC/LTO cell with a Li metal reference electrode after the first charge-discharge cycle between 2.5–4.6 V at C/20 with a 60 h potentiostatic hold at 4.6 V. The model fit to the mid-frequency semicircle data is shown in blue.

The mid-frequency semicircle, which can be attributed to the electrolyte-oxide interfacial impedance,[8,9] was fit using a simple electrochemical equivalent circuit composed of: i) a resistor (R_1) for horizontal displacement of the semicircle, and ii) a resistor (R_2) and a constant phase element (CPE) in parallel to describe the electrolyte-oxide interface resistance. The equivalent circuit is shown below:

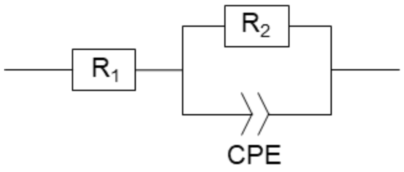

The fit to the data is shown in Figure S9 by a blue line through the red colored data points. Extracted fitting parameter R_2 is plotted as a function of potential and SOC in Figure 4e-f.

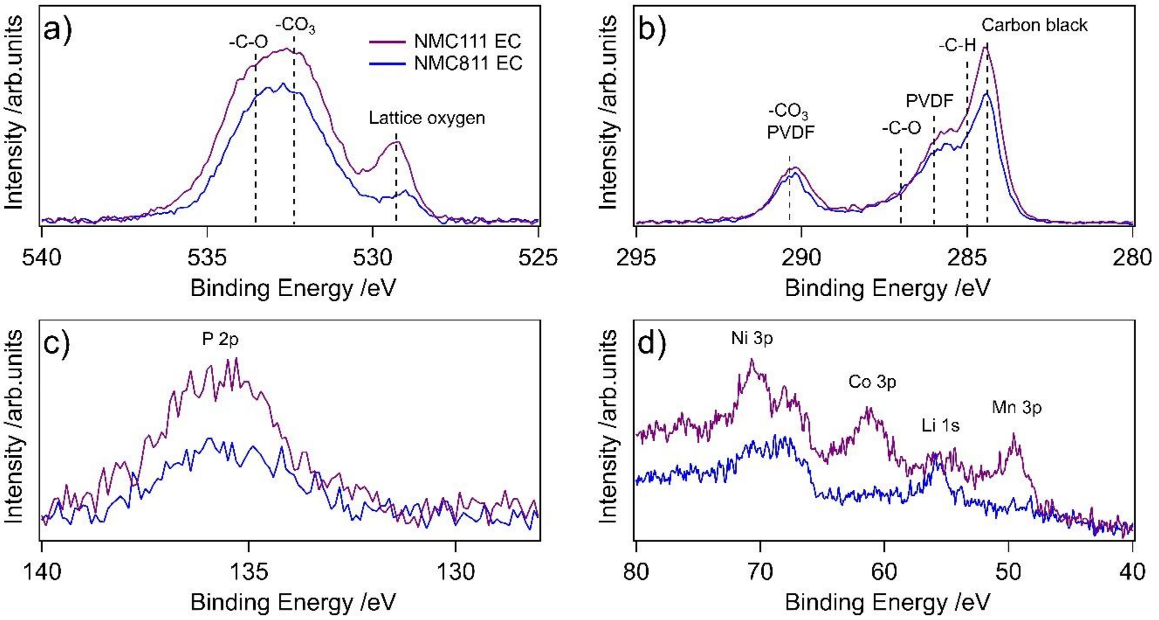

Figure S10. XPS spectra of NMC electrodes extracted from NMC/LTO cells after the first charge-discharge cycle between 1.45–3.05 V at C/20 with a 60 h potentiostatic hold at 3.05 V for NMC111 and 811 with electrolyte 1.5 M $LiPF_6$ in ethylene carbonate (EC). Kimwipe paper (dried under dynamic vacuum at 120 °C) was used as the separator in these cells. a) O 1s spectra. b) C 1s spectra. c) P 2p spectra. d) Ni 3p, Co 3p, Li 1s, and Mn 3p core levels plotted without background subtraction.

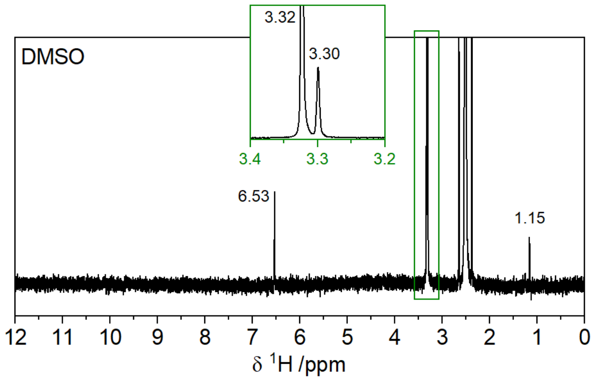

Figure S11. ^{1}H NMR of pristine dimethyl sulfoxide (DMSO). The inset shows a magnified view of the region 3.2-3.4 ppm.

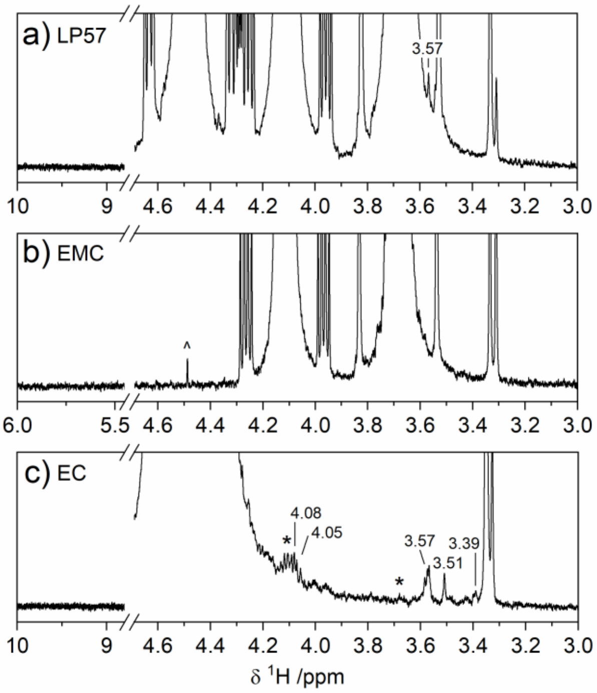

Figure S12. ^{1}H NMR of pristine electrolytes. (a) LP57. (b) 1.5 M $LiPF_6$ in ethyl methyl carbonate (EMC) – the chevron symbol marks a signal from a trace ethylene carbonate (EC) impurity. (c) 1.5 M $LiPF_6$ in EC – the asterisks marks signals from a trace EMC impurity. The signals labelled in (a) and (c) are attributed to hydrolysis products of EC. Those at 4.08 (t) and 3.57 (t) ppm are from lithium ethylene monocarbonate (LEMC), while those at 3.51 and 3.39 ppm are from poly-ethylene oxide (EO) based oligomers and/or ethylene glycol.

Table S5. Peak area fraction $PO_2F_2^-$ / PF_6^- determined from the ^{19}F NMR spectra in Figure 8.

Electrolyte	Cathode	Peak area fraction (×10^3) $PO_2F_2^-$ / PF_6^-
1.5 M $LiPF_6$ in EMC	NMC111	8.81
	NMC811	10.70
1.5 M $LiPF_6$ in EC	NMC111	3.81
	NMC811	12.01

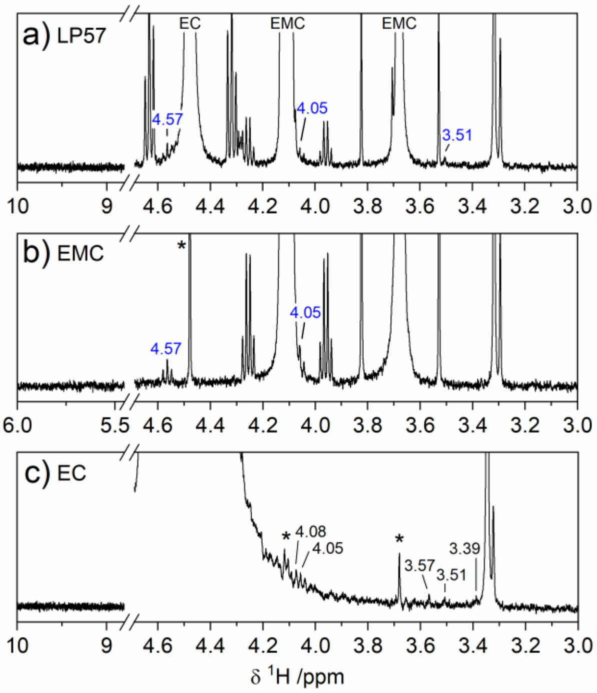

Figure S13. ^{1}H NMR spectra of the electrolyte extracted from LMO/LTO cells after the first charge-discharge cycle between 1.45–3.05 V at C/20 with a 60 h potentiostatic hold at 3.05 V with electrolytes (a) LP57, (b) 1.5 M $LiPF_6$ in ethyl methyl carbonate (EMC), and (c) 1.5 M $LiPF_6$ in ethylene carbonate (EC). Signals of EC and EMC are annotated in (a). Signals of a trace EC impurity in 1.5 M $LiPF_6$ in EMC electrolyte, and a trace EMC impurity in 1.5 M $LiPF_6$ in EC electrolyte, are marked in (b-c) with an asterisk. The chemical shift labels in black are also present in the pristine electrolyte, while blue corresponds to signals that appear after the cycling protocol.

Table S6. The concentration of Ni, Mn, and Co dissolved in the electrolyte and deposited on the LTO anode for NMC111 and 811 and for electrolytes LP57, 1.5 M $LiPF_6$ in ethylene carbonate (EC), and 1.5 M $LiPF_6$ in ethyl methyl carbonate (EMC). The quoted error in parenthesis represents the spread of the ICP-OES measurement from two duplicate cells.

		Ni		Mn		Co	
Cathode	Electrolyte	Electrolyte /ug $g^{-1}_{electrolyte}$	LTO /ug g^{-1}_{LTO}	Electrolyte /ug $g^{-1}_{electrolyte}$	LTO /ug g^{-1}_{LTO}	Electrolyte /ug $g^{-1}_{electrolyte}$	LTO /ug g^{-1}_{LTO}
NMC111	LP57	7(4)	5(5)	1.0(4)	22(2)	0.62(2)	5.8(6)
	1.5 M $LiPF_6$ in EC	2(1)	7(6)	0.4(1)	27(2)	0.3(1)	6.4(7)
	1.5 M $LiPF_6$ in EMC	2(1)	0(1)	0.5(1)	6(1)	0.159(2)	2.3(6)
NMC811	LP57	7(1)	37(2)	0.80(7)	27.0(2)	0.26(2)	2.8(3)
	1.5 M $LiPF_6$ in EC	4.4(4)	194(3)	0.43(3)	48.8(3)	0.14(2)	10.4(4)
	1.5 M $LiPF_6$ in EMC	2.2(3)	16(2)	0.24(6)	7.1(3)	0.21(4)	0.8(2)

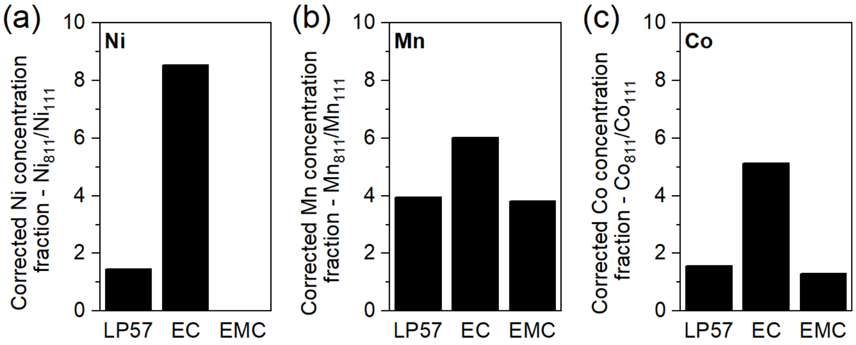

Figure S14. Corrected transition metal (TM) concentration fraction for NMC811 compared to NMC111 (TM_{811}/TM_{111}) for (a) Ni, (b) Mn, and (c) Co dissolved in the electrolyte and deposited on LTO electrodes extracted from NMC/LTO cells after the first charge-discharge cycle between 1.45–3.05 V at C/20 with a 60 h potentiostatic hold at 3.05 V with electrolytes LP57, 1.5 M $LiPF_6$ in ethylene carbonate (EC), and 1.5 M $LiPF_6$ in ethyl methyl carbonate (EMC). The measured TM_{811}/TM_{111} fraction is corrected by dividing the value by the relative fraction of the TM in pristine NMC811 compared to NMC111 – e.g. for Ni the correction factor is 0.8/0.33=2.424 and for Mn and Co the correction factor is 0.1/0.33=0.303.

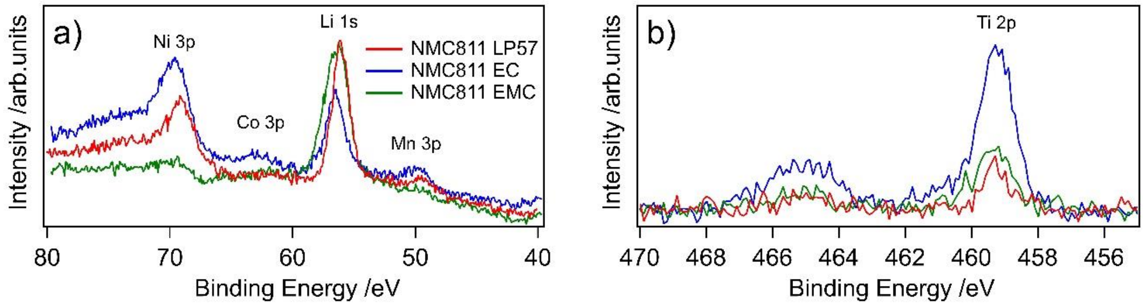

Figure S15. XPS spectra of LTO electrodes extracted from NMC/LTO cells after the first charge-discharge cycle between 1.45–3.05 V at C/20 with a 60 h potentiostatic hold at 3.05 V with NMC811 and electrolytes LP57, 1.5 M $LiPF_6$ in ethyl methyl carbonate (EMC), and 1.5 M $LiPF_6$ in ethylene carbonate (EC). a) Ni 3p, Co 3p Li 1s and Mn 3p core levels plotted without any background subtraction. b) Ti 2p core levels.

Scheme S1: (a-b) Water and (c-d) OH⁻ driven hydrolysis of (a, c) ethylene carbonate (EC) and (b, d) ethyl methyl carbonate (EMC).[10–12]

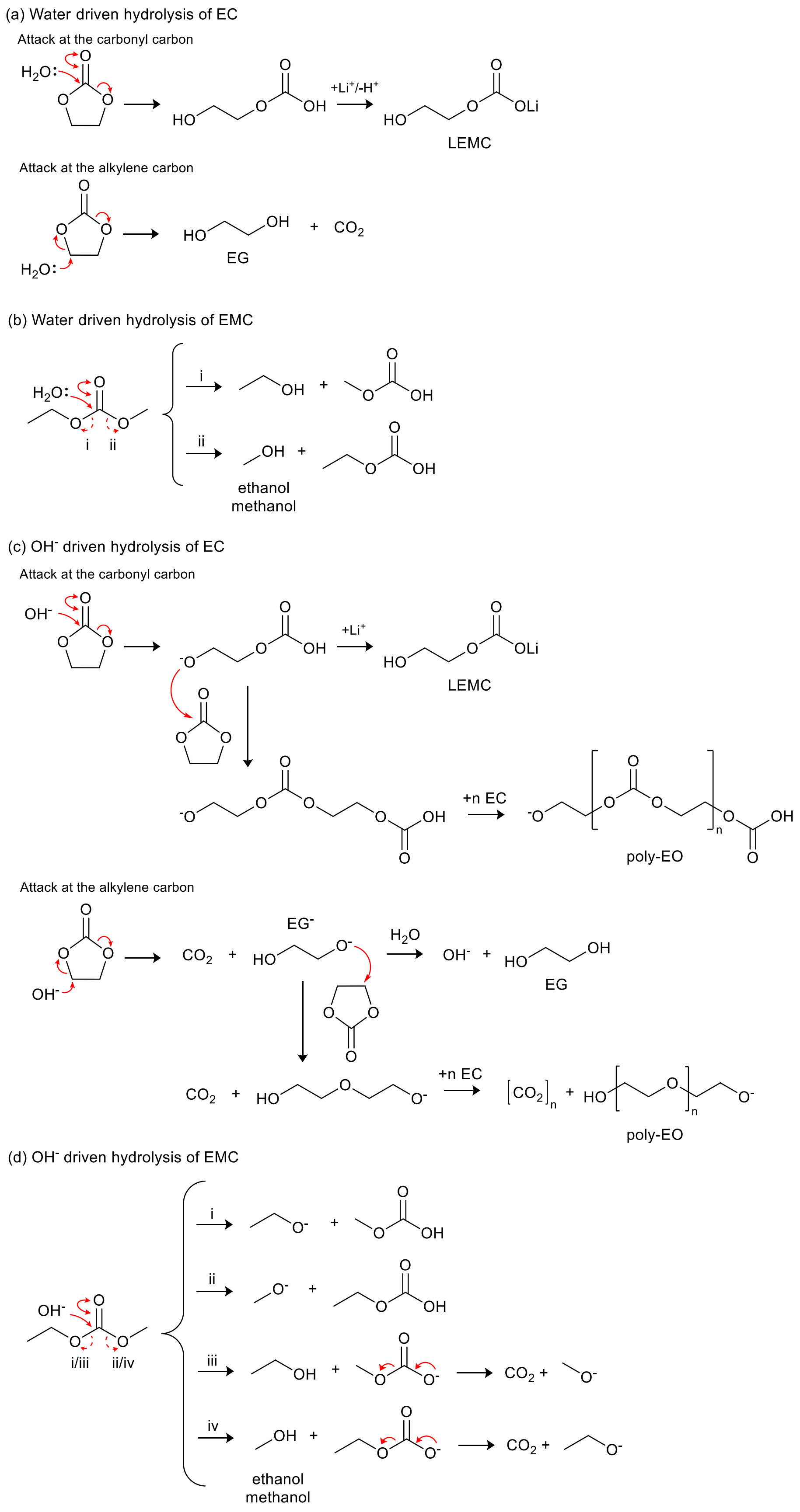

Scheme S2. Electrochemical oxidation of (a) ethylene carbonate (EC)[13,14] and (b) ethyl methyl carbonate (EMC). Reactions for EMC are based on those in Moshkovich et al.[15]

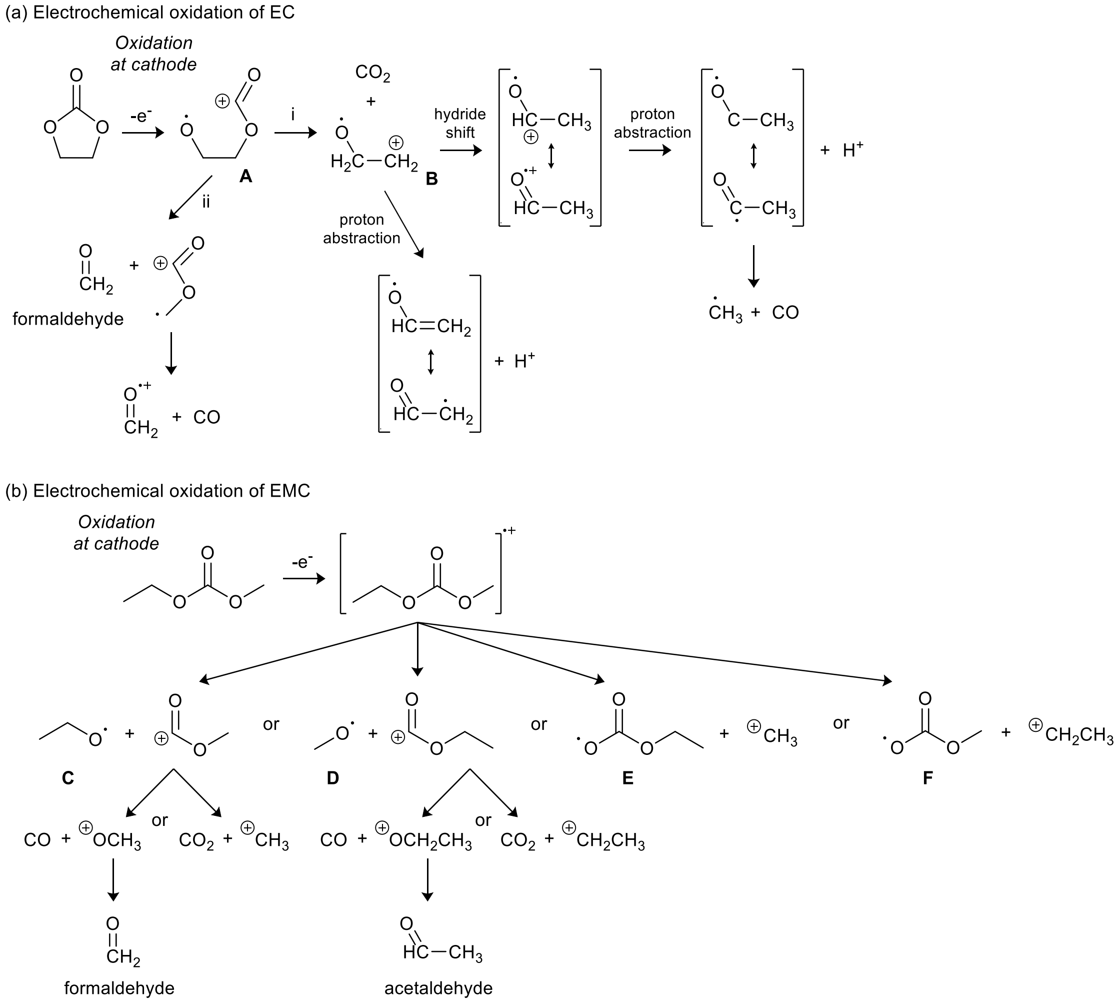

References

(1) He, Y. B.; Li, B.; Liu, M.; Zhang, C.; Lv, W.; Yang, C.; Li, J.; Du, H.; Zhang, B.; Yang, Q. H.; Kim, J. K.; Kang, F. Gassing in $Li_4Ti_5O_{12}$-Based Batteries and Its Remedy. *Sci. Rep.* **2012**, *2* (1), 1–9. https://doi.org/10.1038/srep00913.

(2) Bernhard, R.; Meini, S.; Gasteiger, H. A. On-Line Electrochemical Mass Spectrometry Investigations on the Gassing Behavior of $Li_4Ti_5O_{12}$ Electrodes and Its Origins. *J. Electrochem. Soc.* **2014**, *161* (4), A497–A505. https://doi.org/10.1149/2.013404jes.

(3) Jung, R.; Metzger, M.; Maglia, F.; Stinner, C.; Gasteiger, H. A. Chemical versus Electrochemical Electrolyte Oxidation on NMC111, NMC622, NMC811, LNMO, and Conductive Carbon. *J. Phys. Chem. Lett.* **2017**, *8* (19), 4820–4825. https://doi.org/10.1021/acs.jpclett.7b01927.

(4) Jung, R.; Metzger, M.; Maglia, F.; Stinner, C.; Gasteiger, H. A. Oxygen Release and Its Effect on the Cycling Stability of $LiNi_xMn_yCo_zO_2$ (NMC) Cathode Materials for Li-Ion Batteries . *J. Electrochem. Soc.* **2017**, *164* (7), A1361–A1377. https://doi.org/10.1149/2.0021707jes.

(5) Streich, D.; Erk, C.; Guéguen, A.; Müller, P.; Chesneau, F. F.; Berg, E. J. Operando Monitoring of Early Ni-Mediated Surface Reconstruction in Layered Lithiated Ni-Co-Mn Oxides. *J. Phys. Chem. C* **2017**, *121* (25), 13481–13486. https://doi.org/10.1021/acs.jpcc.7b02303.

(6) Hobold, G. M.; Khurram, A.; Gallant, B. M. Operando Gas Monitoring of Solid Electrolyte Interphase Reactions on Lithium. *Chem. Mater.* **2020**, *32* (6), 2341–2352. https://doi.org/10.1021/acs.chemmater.9b04550.

(7) Fang, C.; Li, J.; Zhang, M.; Zhang, Y.; Yang, F.; Lee, J. Z.; Lee, M. H.; Alvarado, J.; Schroeder, M. A.; Yang, Y.; Lu, B.; Williams, N.; Ceja, M.; Yang, L.; Cai, M.; Gu, J.; Xu, K.; Wang, X.; Meng, Y. S. Quantifying Inactive Lithium in Lithium Metal Batteries. *Nature* **2019**, *572* (7770), 511–515. https://doi.org/10.1038/s41586-019-1481-z.

(8) Dees, D.; Gunen, E.; Abraham, D.; Jansen, A.; Prakash, J. Alternating Current Impedance Electrochemical Modeling of Lithium-Ion Positive Electrodes. *J. Electrochem. Soc.* **2005**, *152* (7), A1409. https://doi.org/10.1149/1.1928169.

(9) Gilbert, J. A.; Bareño, J.; Spila, T.; Trask, S. E.; Miller, D. J.; Polzin, B. J.; Jansen, A. N.; Abraham, D. P. Cycling Behavior of NCM523/Graphite Lithium-Ion Cells in the 3–4.4 V Range: Diagnostic Studies of Full Cells and Harvested Electrodes. *J. Electrochem. Soc.* **2017**, *164* (1), A6054–A6065. https://doi.org/10.1149/2.0081701jes.

(10) Lee, J. C.; Litt, M. H. Ring-Opening Polymerization of Ethylene Carbonate and Depolymerization of Poly(Ethylene Oxide-Co-Ethylene Carbonate). *Macromolecules* **2000**, *33* (5), 1618–1627. https://doi.org/10.1021/ma9914321.

(11) Metzger, M.; Strehle, B.; Solchenbach, S.; Gasteiger, H. A. Hydrolysis of Ethylene Carbonate with Water and Hydroxide under Battery Operating Conditions. *J. Electrochem. Soc.* **2016**, *163* (7), A1219–A1225. https://doi.org/10.1149/2.0411607jes.

(12) Barnes, P.; Smith, K.; Parrish, R.; Jones, C.; Skinner, P.; Storch, E.; White, Q.; Deng, C.; Karsann, D.; Lau, M. L.; Dumais, J. J.; Dufek, E. J.; Xiong, H. A Non-Aqueous Sodium Hexafluorophosphate-Based Electrolyte Degradation Study: Formation and Mitigation of Hydrofluoric Acid. *J. Power Sources* **2020**, *447*, 227363. https://doi.org/10.1016/j.jpowsour.2019.227363.

(13) Xing, L.; Li, W.; Wang, C.; Gu, F.; Xu, M.; Tan, C.; Yi, J. Theoretical Investigations on Oxidative Stability of Solvents and Oxidative Decomposition Mechanism of Ethylene Carbonate for Lithium Ion Battery Use. *J. Phys. Chem. B* **2009**, *113* (52), 16596–16602. https://doi.org/10.1021/jp9074064.

(14) Metzger, M.; Strehle, B.; Solchenbach, S.; Gasteiger, H. A. Origin of H_2 Evolution in LIBs: H_2O Reduction vs. Electrolyte Oxidation . *J. Electrochem. Soc.* **2016**, *163* (5), A798–A809. https://doi.org/10.1149/2.1151605jes.

(15) Moshkovich, M.; Cojocaru, M.; Gottlieb, H. E.; Aurbach, D. The Study of the Anodic Stability of Alkyl Carbonate Solutions by in Situ FTIR Spectroscopy, EQCM, NMR and MS. *J. Electroanal. Chem.* **2001**, *497* (1–2), 84–96. https://doi.org/10.1016/S0022-0728(00)00457-5.
